# Bright fluorescent silica-nanoparticle probes for high-resolution STED and confocal microscopy

**DOI:** 10.3762/bjnano.8.130

**Published:** 2017-06-21

**Authors:** Isabella Tavernaro, Christian Cavelius, Henrike Peuschel, Annette Kraegeloh

**Affiliations:** 1 INM - Leibniz Institute for New Materials, Nano Cell Interactions Group, Campus D2 2, D-66123 Saarbrücken, Germany; 2 nanoSaar Lab GmbH, Comotorstr. 2, D-66802 Überherrn, Germany

**Keywords:** bioimaging, confocal microscopy, multistep synthesis approach, organic fluorescent dyes, silica nanoparticles, stimulated emission depletion (STED) microscopy

## Abstract

In recent years, fluorescent nanomaterials have gained high relevance in biological applications as probes for various fluorescence-based spectroscopy and imaging techniques. Among these materials, dye-doped silica nanoparticles have demonstrated a high potential to overcome the limitations presented by conventional organic dyes such as high photobleaching, low stability and limited fluorescence intensity. In the present work we describe an effective approach for the preparation of fluorescent silica nanoparticles in the size range between 15 and 80 nm based on L-arginine-controlled hydrolysis of tetraethoxysilane in a biphasic cyclohexane–water system. Commercially available far-red fluorescent dyes (Atto647N, Abberior STAR 635, Dy-647, Dy-648 and Dy-649) were embedded covalently into the particle matrix, which was achieved by aminosilane coupling. The physical particle attributes (particle size, dispersion, degree of agglomeration and stability) and the fluorescence properties of the obtained particles were compared to particles from commonly known synthesis methods. As a result, the spectroscopic characteristics of the presented monodisperse dye-doped silica nanoparticles were similar to those of the free uncoupled dyes, but indicate a much higher photostability and brightness. As revealed by dynamic light scattering and ζ-potential measurements, all particle suspensions were stable in water and cell culture medium. In addition, uptake studies on A549 cells were performed, using confocal and stimulated emission depletion (STED) microscopy. Our approach allows for a step-by-step formation of dye-doped silica nanoparticles in the form of dye-incorporated spheres, which can be used as versatile fluorescent probes in confocal and STED imaging.

## Introduction

With the emergence of nanotechnology, numerous applications have been developed using the size-related properties of nanoparticles [1–2]. In biomedical research, nanoparticles are applied as drug carriers [3–5], as transfection agents [6–7], for cancer treatment by local hyperthermia [8–9], for labelling [10–11] and for bioimaging [12–14]. The detection of cell-associated and internalised nanoparticles and the analysis of their interactions with extracellular or subcellular components and structures are not only important in the nanomedical field but also in nanotoxicology. Due to the fact that more and more nano-based materials come to market and are developed for everyday use, the latter discipline is targeting the effects of engineered nanomaterials on living organisms [15–17]. In order to localise nano-sized materials inside cells, various imaging techniques are available nowadays, each exhibiting intrinsic limitations. Conventional fluorescence-based methods, for example, are not applicable for spatially-resolved detection of nano-scaled structures, but, on the other hand, are suitable for observing dynamic processes using living cells [18–20]. In addition, the development of novel so-called “super-resolution” optical imaging techniques allows for the imaging of objects beyond the diffraction limit [21–22]. For example, stimulated emission depletion (STED) microscopy utilises the saturable deexcitation of fluorophores within an excited volume using a second laser with a red-shifted wavelength. Typically, a donut-shaped depletion laser profile is used to enhance lateral resolution, through focal laser intensities in the range of 100–500 MW·cm^−2^ [23]. All in all, STED microscopy requires fluorescently labelled markers with high photostability to identify relevant interactions between nanoparticles and cellular structures.

In the last years different types of markers have been used for fluorescence-based microscopy, such as modified inorganic gold nanoparticles [24–25], silica nanoparticles [26] or quantum dots [27–28]. These fluorescent nanoparticles fulfil some of the specific requirements and overcome disadvantages of common organic fluorescent dyes [29]. Especially silica nanoparticles have proven useful, since silicon chemistry provides a versatile tool for the modification of the silica surface with a variety of functional groups [30] or biomolecules [31]. This material also allows for the incorporation of dyes into the particle matrix without changing their optical properties [32]. However, the incorporation of fluorescent dyes into the silica matrix, which is mostly based on non-polar aromatic or conjugated π-electron systems [33], is not easy to achieve due to the hydrophobic character of the organic dye molecules.

Commonly, fluorescent silica nanoparticles are obtained by hydrolysis and condensation of a silica precursor via sol–gel or reverse microemulsion synthesis [34]. Using the traditional sol–gel synthesis of silica particles described by Stöber et al. [35], particles are available in the size range of a few tens of nanometres up to the micrometre range. Although the Stöber method can be utilised to obtain particles with a narrow size distribution in the range of 100 nm to several micrometres, the method is less suited for the synthesis of silica nanoparticles with a narrow dispersity in the size regime below 100 nm. Thus many research studies have been based on this approach, developing new synthesis methods of fluorescence silica nanoparticles [36–38]. One of the most outstanding methods was developed by Wiesner et al., who designed new small fluorescent core–shell silica nanoparticles [39–41]. These so called “Cornell dots” (C-dots) have recently been approved as an "investigational new drug" (IND) by the U.S. Food and Drug Administration (FDA) and are being tested in human clinical trials [42]. Although many other reports about the preparation of silica nanoparticles with a fluorescent label have been published [43], the synthesis of silica nanoparticles in the lower nanoscale range with a narrow size distribution and sufficient dye labelling remains a challenge.

In previous works, we presented fluorescent 30 nm, 85 nm and 125 nm large silica nanoparticles with covalently attached Atto647N dye, which was either coupled to the particle surface or into the silica matrix. These fluorescent nanoparticles were suitable for biological nanoparticle uptake experiments and have been used to determine the intracellular migration and nuclear penetration after uptake into Caco-2 cells [44]. They have also been used to analyse their intracellular agglomeration and their association with intracellular vesicles in living A549 cells, as well as to quantify the number of internalised nanoparticles in these cells [45–47]. In another study small silica nanoparticles with diameters of 25 and 40 nm and modified with Atto647N dye were used to investigate the uptake in macrophages [48].

Herein we present the improved syntheses of the covalently incorporated (fully dyed) fluorescent silica nanoparticles in more detail by describing the syntheses of the dye–APTES conjugates, the different synthesis steps, a comparison with commonly used methods and variations of the used dyes.

## Results and Discussion

Our main goal was the synthesis of monodisperse silica nanoparticles in the size range of 15 to 80 nm and their modification with fluorescent NIR dyes to enable stimulated emission depletion (STED) and confocal imaging. For this purpose, we adopted a literature-known synthesis of silica nanoparticles using an L-arginine-catalysed hydrolysis of tetraethoxysilane (TEOS) in a biphasic water–cyclohexane system [49] and incorporated different fluorescent NIR dyes into the silica particle matrix.

### Fully dyed (FD) silica nanoparticles through embedding of Atto647N

**Scheme 1:**
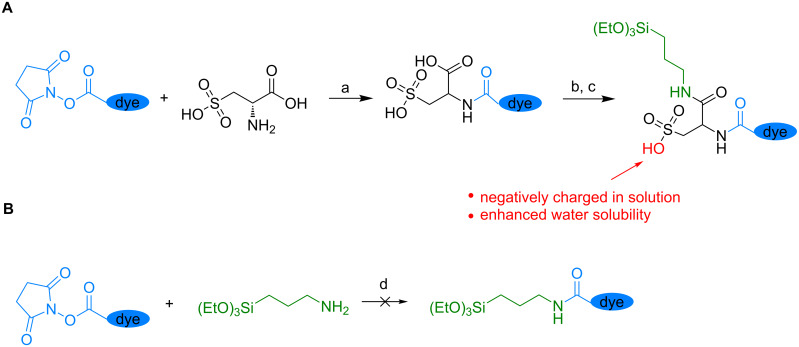
Pre-synthesis modification steps of the Atto647N dye. Path A: The dye was modified with a cysteic acid linker–APTES conjugate (a: DMSO, NEt_3_, rt, overnight; b: DMSO, EDC, NHS, rt, 30 min; c: DMSO, APTES, rt, overnight). A modification of Atto647N with APTES only (d: DMSO, APTES, rt, 2 h) was not possible (path B).

For application in STED microscopy the dye-doped silica nanoparticles have to fulfil some criteria. First of all, an efficient dye excitation in the range of its absorption maximum has to be achieved. In this study, a pulsed diode laser with a wavelength of 635 nm was used for dye excitation. Secondly, a significant stimulated-emission cross section is needed for effective depletion by the STED laser. In a typical STED implementation, available to a broad range of users, this is achieved in the range between 750 and 800 nm using a tuneable Ti–sapphire laser. For this reason, one of the most frequently used red-emitting dyes for STED microscopy, the commercially available carbopyronine dye Atto647N was selected as a model dye [50]. It matches the preferred spectral characteristics and has gained popularity as fluorescence label due to its strong absorption, excellent fluorescence, quantum yield and good solubility [51–52]. However, Atto647N had to be modified before embedding into the silica particle matrix (Scheme 1). A cysteic acid spacer was introduced between dye and (3-aminopropyl)triethoxysilane (APTES) linker to provide negatively charged sulfonic acid groups (Scheme 1A). This pre-synthesis modification step was necessary, since attempts to incorporate sufficient amounts of Atto647N into the silica matrix via exclusive APTES linkage were not successful (Scheme 1B). During the course of synthesis a drastically increased dispersity of the particles could be observed. In cases of higher Atto647N concentrations, dye-containing blue gels were even obtained at the bottom of the flask. This may be attributed to the positive charge of the Atto647N dye molecule after embedding, leading to a reduction of the negative ζ-potential of the silica surface and thus to agglomeration of the particles. However, introduction of cysteic acid to the side chain of the dye increased the hydrophilic character of the dye avoiding agglomeration and gel formation during the synthesis.

**Figure 1:**
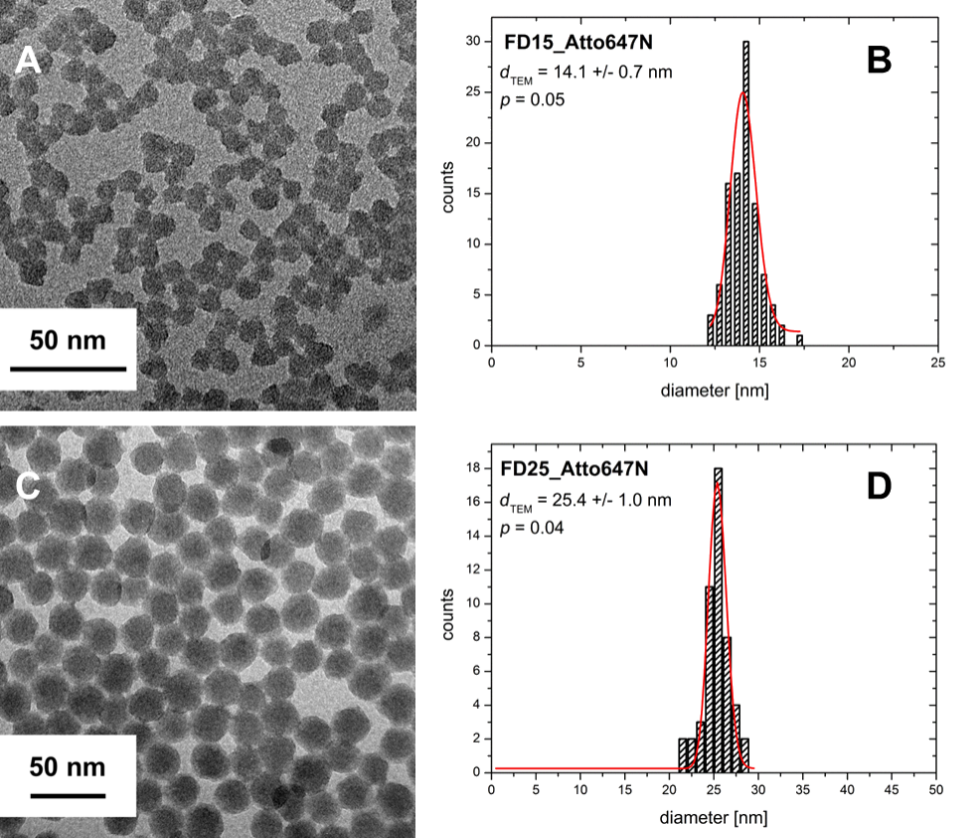
TEM images and size histograms of the Atto647N-doped silica nanoparticles. A: TEM image of FD15_Atto647N particles; B: size histogram derived from the TEM image of FD15_Atto647N; C: TEM image of FD25_Atto647N particles; D: size histogram derived from the TEM image of FD25_Atto647N.

By using the Atto647N–cysteic acid–APTES conjugate (Atto647N-CS-APTES) and a precise adjustment of the reaction parameters, monodisperse silica nanoparticles with a mean particle diameter of 15 nm (5% size distribution; FD15_Atto647N) or 25 nm (4%; FD25_Atto647N) could be obtained (Figure 1). Hydrodynamic diameters determined by dynamic light scattering (DLS) were in accordance with diameters obtained from SEM or TEM image analysis (Table 1). These results show that no agglomerates of the nanoparticles exist in the samples. Further, the aqueous nanoparticle suspensions exhibit a negative ζ-potential of −15 to −21 mV. Due to electrostatic repulsion that leads to a high stability against agglomeration neither significant changes of the agglomeration state nor aggregation were observed over 12 months storage.

**Table 1:** Overview of the physical particle attributes (size, degree of dispersion, and charge) of the synthesised Atto647N-dyed silica nanoparticles. To determine the particle diameter and the size distribution, the samples were characterised by TEM image analysis. The hydrodynamic diameter of the particles in H_2_O was measured by DLS, whereas the particle charge was determined by ζ-potential measurements. The mean hydrodynamic diameter of the particles was determined, using the “volume distribution”. Numbers in brackets indicate the size dispersity *p*. It is calculated with the formula *p* = σ/μ with σ being the standard deviation and μ being the mean value. n.d. = not determined.

SiO_2_ NP	particle diameter, *d*_TEM_ [nm]	hydrodynamic diameter, *d*_h_ [nm]	ζ-potential [mV]

FD15_Atto647N	14.1 ± 0.7 (0.05)	14 ± 3 (0.19)	−15.3
FD25_Atto647N	25.4 ± 1.0 (0.04)	26 ± 5 (0.18)	−20.9
FD45_Atto647N	50.0 ± 1.8 (0.04)	47 ± 10 (0.22)	−37.5
FD60_Atto647N	66.5 ± 2.2 (0.03)	65 ± 14 (0.22)	−43.3
FD80_Atto647N	83.6 ± 2.0 (0.02)	92 ± 19 (0.21)	−43.3

Me15	13.8 ± 0.5 (0.04)	n.d.	−28.9
Me25	22.9 ± 0.9 (0.04)	23 ± 4 (0.17)	−30.0
Me45	40.7 ± 2.7 (0.07)	44 ± 9 (0.21)	−23.5
Me60	62.1 ± 2.2 (0.04)	62 ± 13 (0.21)	−39.1
Me80	80.4 ± 2.2 (0.03)	84 ± 15 (0.18)	−35.4

**Figure 2:**
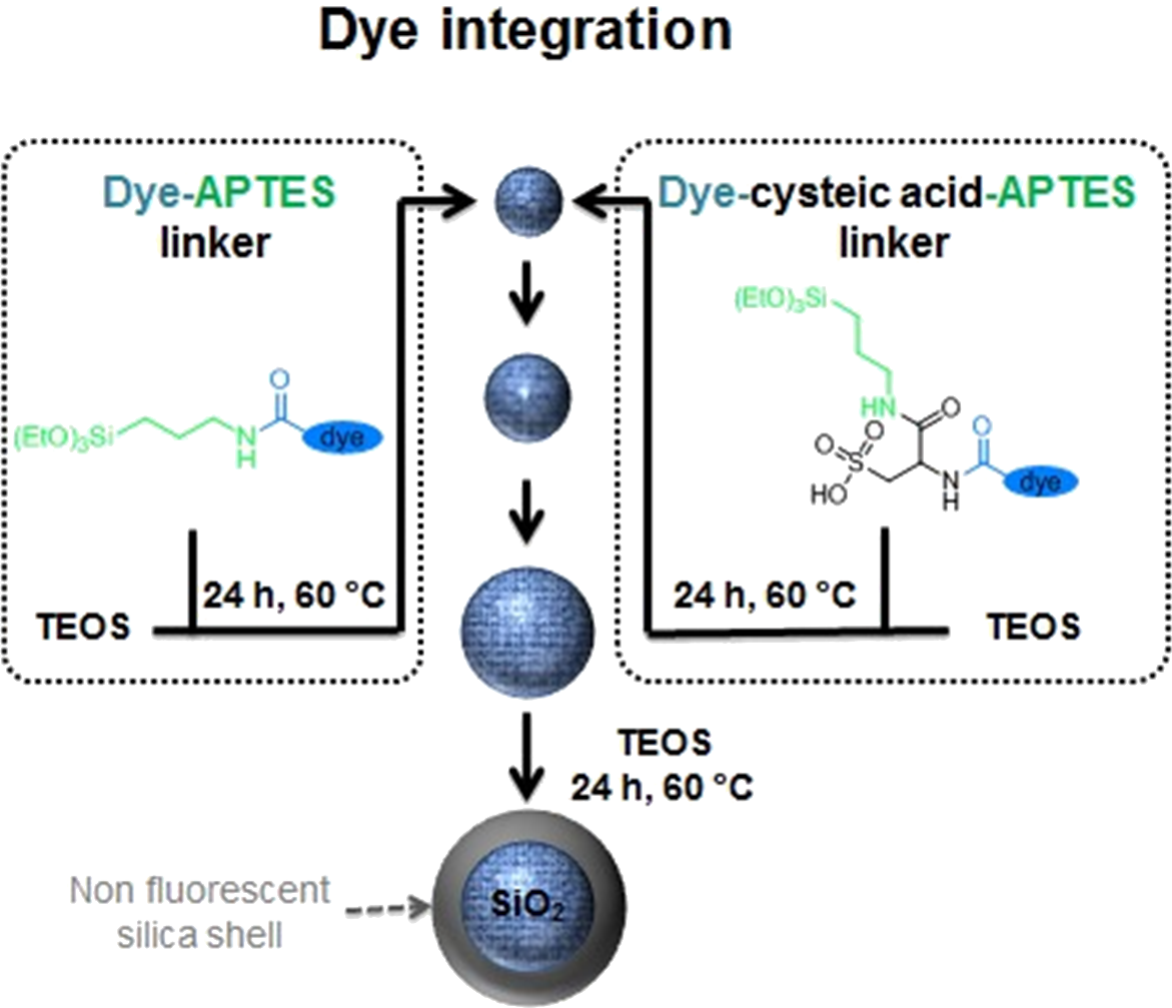
Schematic synthetic procedures for the multistep preparation of dye-conjugated silica nanoparticles in the size range between 15 and 80 nm. All particle growth steps were conducted via L-arginine-catalysed hydrolysis of TEOS in a biphasic water–cyclohexane system at 60 °C. Dye embedding was achieved during particle (re)growth by addition of modified dye. Dyomics dyes and the dye Star635 could be directly incorporated into the particle matrix if coupled to APTES. For the dye Atto647N, the introduction of a negatively charged cysteic acid group is required to yield well-defined fluorescent probes. If necessary the obtained silica nanoparticles can be further covered by a pure silica layer through repeating the shell growth step without embedding of fluorescent dyes.

Larger particles were synthesised by multiple regrowth steps of the FD25 nanoparticles (Figure 2). A comparison of the mean particle diameters after each regrowth step by electron microscopy, indicate a particle growth of 15–20 nm per step. Even though the hydrodynamic diameters of the particles were slightly higher than the values obtained by statistical SEM and TEM image analysis (Table 1), they confirmed the observed trend. All particles exhibited a negative ζ-potential that slightly decreased with increasing particle size. These measured ζ-potentials were in accordance with the ζ-potential values determined for pure silica nanoparticles of similar size (Table 1). In addition, a comparison of Atto647N-dyed silica nanoparticles with pure silica nanoparticles (Me series) shows nearly constant particle sizes with narrow size distributions. Therefore, a significant influence of Atto647N–CS–APTES on the particle synthesis and the physical particle attributes can be excluded.

### Comparison of FD particles with Stöber synthesis and C-dots synthesis

Furthermore, the above presented Atto647N-doped silica nanoparticles were compared to silica nanoparticles of other literature-known synthesis methods. For this purpose different sizes of Atto647N-doped silica nanoparticles were synthesised following either the method of Stöber [36] or the C-dots method [42]. Both methods utilise ammonia as a catalyst, which has a large influence on the dimension and morphology of the obtained silica nanoparticles. Indeed, it is well known that the particle size increases with an increase in ammonia concentration [53–54]. The use of ammonia could be a major limitation for dyes, which are sensitive to high-alkaline pH values. Another drawback of both methods is a broader particle size distribution (Table 2). The Stöber method yielded nanoparticles with a mean particle size of 16 and 64 nm and a dispersity of 12% and 13% respectively, whereas the C-dots synthesis revealed particles with a mean size of 12 nm (7%). Compared to our presented method the achieved dispersity was at least twice as high for the Stöber method and approximately identically for the C-dots method (Table 2). The shape of the 15 nm large silica nanoparticles, obtained by the Stöber synthesis and the C-dots method, are more irregular and less spherical than FD15_Atto647N (Figure S15, Supporting Information File 1). Once again, no significant influence of the dye embedding during the particle synthesis could be observed.

**Table 2:** Overview of the physical particle attributes of Atto647N-dyed silica nanoparticles, synthesised by the Stöber method or the C-dots method. Particle diameter and size distribution of the samples were characterised by TEM image analysis. The hydrodynamic diameter of the particles and their state of agglomeration in H_2_O was measured by DLS, whereas the particle charge was determined by ζ-potential measurements. The mean hydrodynamic diameter of the particles was determined, using the “volume distribution”. Numbers in brackets indicate the size dispersity *p*.

SiO_2_ NP	particle diameter, *d*_TEM_ [nm]	hydrodynamic diameter, *d*_h_ [nm]	ζ-potential [mV]
	
Stoe15_Atto647N	16.3 ± 2.1 (0.13)	27 ± 6 (0.23)	−28.7
Stoe75_Atto647N	69.5 ± 7.0 (0.10)	75 ± 16 (0.21)	−42.4
CD_Atto647N	11.5 ± 0.7 (0.06)	12 ± 3 (0.25)	−45.0

Stoe15	15.6 ± 2.1 (0.13)	19 ± 3 (0.16)	−26.2
Stoe75	65.9 ± 8.4 (0.13)	76 ± 18 (0.24)	−39.6
CD	n.d.	12 ± 3 (0.25)	−29.0

### Variation of the linker, the dye molecules and the dye concentration

To study if either the charge of the used dye or the cysteic acid linker have an influence on the particle synthesis, we chose the neutral dye Abberior STAR 635 (Star635, charge: 0) and three negatively charged pentamethine based Dyomics dyes (Dy-647, charge: −1; Dy-648, charge: −2; Dy-649, charge: −3), which exhibit a high water solubility mainly caused by negatively charged sulfonate side groups (supplier information). Compared to Atto647N, all four dyes could be directly incorporated into the silica matrix with an APTES linker (Figure 2), which supports our hypothesis of the positively charged Atto647N dye. The particle size, the size distribution and the state of agglomeration of the different dye–APTES-conjugated silica nanoparticles were determined after the synthesis by measurements of the ζ-potential and analysis of TEM or SEM images (Table 3). The results indicate a strong dependence of the particle size and nanoparticle dispersity from the chosen dye and its charge. The neutral Star635–APTES conjugate and the highly negatively charged Dy649–APTES conjugate resulted in approximately similar particle sizes compared to FD25_Atto647N, whereas FD_Dy647-APTES and FD_Dy648-APTES indicated significantly larger particle diameters. The synthesis of larger silica nanoparticles showed that the choice of dye had no influence on the particle diameters of the regrowth steps, but on the size distribution.

**Table 3:** Overview of the physical particle attributes of dye–APTES-modified silica nanoparticles. Particle diameter and size distribution of the samples were characterised by SEM image analysis and the particle charge was determined by ζ-potential measurements. Numbers in brackets indicate the size dispersity *p*. n.d. = not determined.

dyed SiO_2_ NP	net charge of dye [*e*]	particle diameter, *d*_SEM_	ζ-potential [mV]

FD_APTES-Dy647	−1	39 ± 11 (0.28)	n.d.
FD_APTES-Dy648	−2	40 ± 12 (0.30)	−20.7
FD_APTES-Dy649	−3	29 ± 8 (0.28)	−26.1
FD_APTES-Star635	0	33 ± 3 (0.08)^a^	−28.0

FD60_APTES-Dy648	−2	60 ± 8 (0.13)	−36.0
FD80_APTES-Dy648	−2	80 ± 8 (0.10)	−40.0

^a^Particle diameter was determined by TEM image analysis.

The synthesized 25 nm large Star635-embedded silica nanoparticles, using the dye modified with cysteic acid, exhibited a mean particle diameter of 20 nm (FD25_Star635) with a narrow size distribution of 4% (Table 4). The particle size distribution is much narrower than the one of the silica nanoparticles modified with the Star635–APTES–conjugate. This allows the conclusion that the cysteic acid modification of the dye–APTES conjugates with its higher level of negative charges had a positive influence on the particle growth independently of the used dye.

**Table 4:** Overview of the physical particle attributes of Star635–CS-modified silica nanoparticles using different dye concentrations during the synthesis. Numbers in brackets indicate the size dispersity *p*.

Star635-modified SiO_2_ NP	added volume of dye, *V* [µL]	particle diameter, *d*_TEM_ [nm]	hydrodynamic diameter, *d*_h_ [nm]	ζ-potential [mV]

FD25_Star635	56.4	19.1 ± 0.8 (0.04)	21 ± 4 (0.18)	−31.4
FD45_Star635	56.4	35.0 ± 1.2 (0.04)	36 ± 7 (0.19)	−43.4
FD60_Star635	56.4	48.1 ± 2.4 (0.05)	49 ± 14 (0.30)	−35.9

FD25_Star635	112.8	24.8 ± 1.3 (0.05)	23 ± 5 (0.21)	−30.6
FD45_Star635	112.8	43.6 ± 2.9 (0.07)	37 ± 9 (0.25)	−39.3
FD60_Star635	112.8	69.6 ± 1.9 (0.03)	80 ± 18 (0.22)	−41.8

In addition, we varied the dye concentration during the multiple synthesis steps to verify its influence on the particle size, dispersity and degree of agglomeration (Table 4). The results of the characterisation by TEM images, DLS and ζ-potential measurements indicate neither an influence on the particle size distribution nor on the degree of agglomeration. Only the particle size increase slightly with an increasing dye concentration Therefore, our synthesis allows one to tune the dye content that can be incorporated into the particle matrix. This is important for cell interaction studies in which a possible influence of the dye on toxicity and cell interaction should be minimised.

### Absorption and fluorescence properties of the dye-doped silica particles

After characterisation of the physical particle attributes, fluorescence and absorption measurements of the dye-doped silica nanoparticles were performed. First of all, we studied the influence of the integration of the dyes into the silica matrix on the absorption and fluorescence signal. Comparison of the UV–vis absorption and fluorescence spectra of dye-doped silica nanoparticles and those of free dye molecules indicated no influence of the integration. The spectra seem to be approximately the same (less than a few nanometres peak shift to longer wavelengths), showing two maxima for the Atto647N dye (Figure S16, Supporting Information File 1) in the absorption spectra, whereas the fluorescence spectra of the dye-modified silica nanoparticles and the free dye molecules indicate a nearly similar maximum at 660 nm. Identical results were obtained for the comparison of the free other dyes with the embedded ones (data not shown). Also a comparison of the UV–vis absorption and fluorescence spectra of the synthesised dye-embedded silica particles after the different regrowth steps (Figure S17, Supporting Information File 1) or the use of different dye concentrations during the individual synthesis steps (Figure S18, Supporting Information File 1) show no influence on the maxima of the spectra.

To demonstrate the stable embedding of the dyes into the silica matrix leaching experiments were performed (Figure S19, Supporting Information File 1). The dye leaching of the particles was investigated by comparing the relative fluorescence intensity before and after centrifugation of the particle suspension through two different membranes (30 kDa and 100 kDa). The measurements revealed almost no dye leaching (less than 10%) the particle suspensions, indicating a more stable embedding than with the Stöber method (14% (15 nm), 34% (60 nm)).

**Figure 3:**
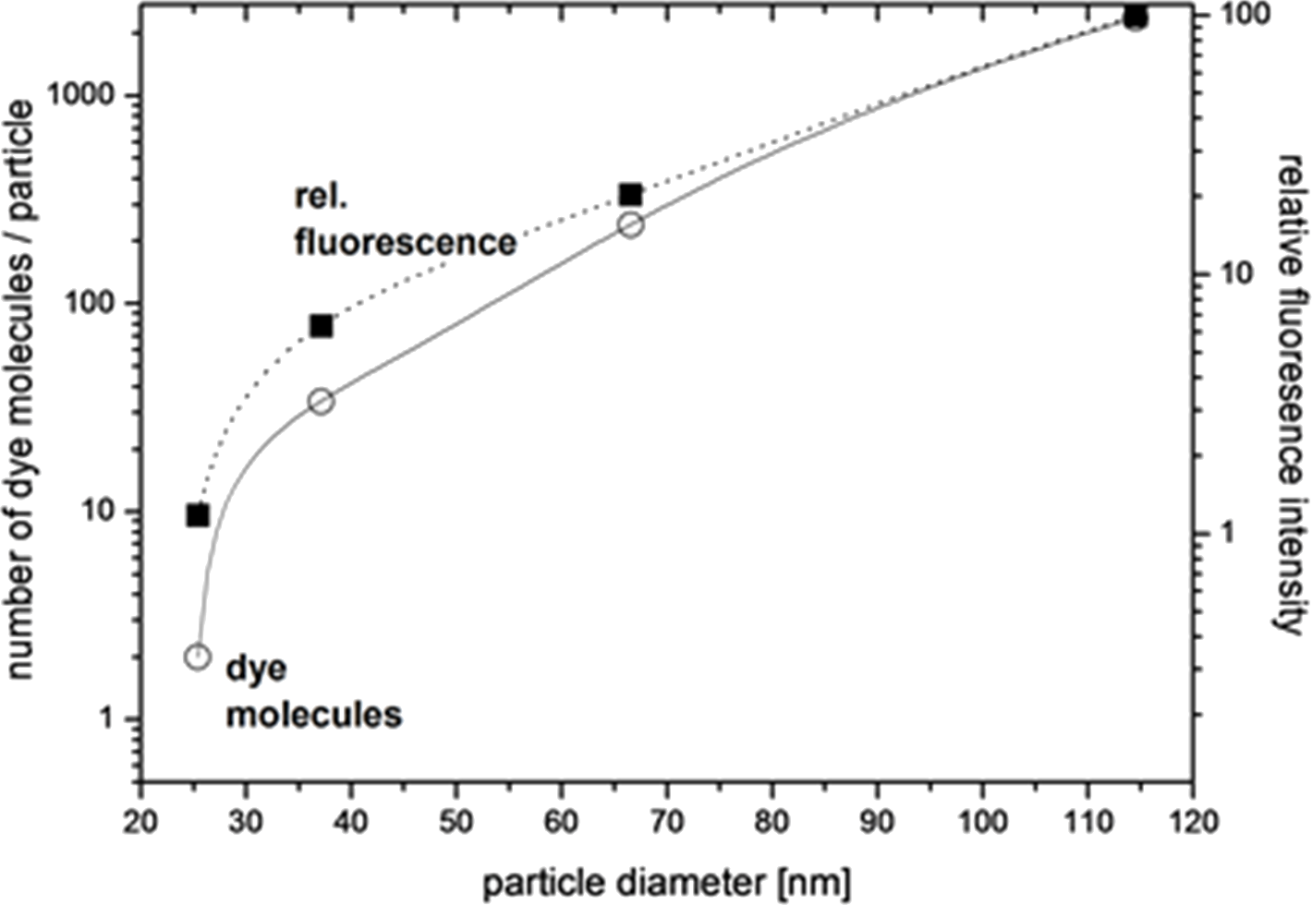
Calculated number of dye molecules per silica particle as a function of size (filled square) and relative fluorescence intensity (open circles) as a function of the particle size. The ratio of fluorophore per particle was calculated based on the concentration of nanoparticles and the molar content of dye assuming a dye immobilisation yield of 100%. Dye-labelled particles show a volume-dependent increase in fluorescence intensity. The amount of immobilised dye can (statistically) be controlled between two (FD25) and several hundred (FD80) dye molecules per particle, just by addition of dye during each regrowth step.

Based on these results, we calculated the dye content of our particles depending on the amount of dye that was employed for particle synthesis, and compared the resulting fluorescence intensity (Figure 3). It turned out, that the number of dye molecules increases as a function of particle size from two molecules per particle (25 nm) to several hundred molecules per particle (80 nm). Thereby the number of incorporated dye molecules can be controlled by the amount of the modified dye and by growing non-fluorescent or fluorescent shells.

### Brightness and quantum yields

**Figure 4:**
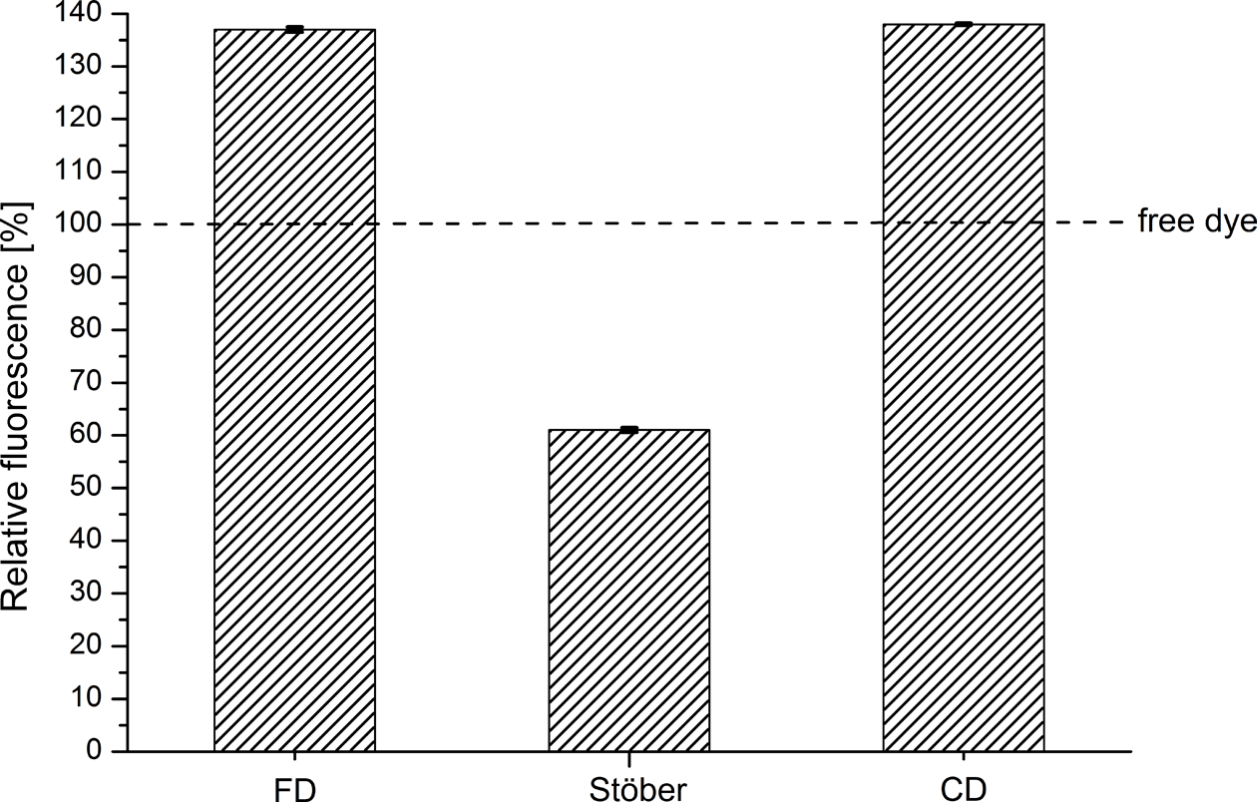
Comparison of the relative fluorescence intensity of FD15_Atto647N, Stoe15_Atto647N and CD_Atto647N. All samples were diluted to an absorption intensity of *A* = 0.012 to ensure an identical number of dye molecules. The fluorescence intensity of free Atto647N–CS–APTES was set to 100%. FD15_Atto647N and CD_Atto647N exhibited a significantly higher relative fluorescence intensity, whereas Stoe15_Atto647N was much less brighter (60%).

Sensitive imaging applications, such as live-cell imaging, three-dimensional imaging or STED microscopy, require a high brightness of the imaging probes [55]. To compare the brightness of the nanoparticles obtained by the different synthetic procedures, three samples with identical particle concentration (0.02 nmol/L) were prepared and the fluorescent intensity was determined. The results indicated that the relative fluorescent of FD15_Atto647N was significantly higher than the one received from Stoe15_Atto647N and CD_Atto647N. This demonstrates once again a larger quantity of embedded dye molecules. Therefore, brightness measurements were repeated with samples indicating the same absorption intensities to ensure the identical number of dye molecules (Figure 4).

In addition, the quantum yields were determined. For the quantum yield measurements the comparative method of Williams et al. [56] was utilized, which involves the use of a well characterised standard with a known quantum yield (Φ_F_) and fitting absorption and emission spectra. Normally, this method cannot be used for samples that exhibit significant scattering since the additional light emission leads to an overestimation of the quantum yield [57]. Nevertheless, the method can be still applied if a scattering correction is performed with the reference sample [58]. In our case, the correction was carried out by incorporating an adequate proportion of silica particles with the same size containing no dye into the reference solution. A diluted sample in water of free Atto647N dye was selected as reference material. In a first round of the quantum yield measurements we studied the influence of the cysteic acid linking to Atto647N. Although a drop of the quantum yield from 65% to 48% could be observed through the linking, the measurements with FD60_Atto647N yielded with 44% nearly the same decrease, which indicated no significant quenching of the embedded dyes.

### Photostability

**Figure 5:**
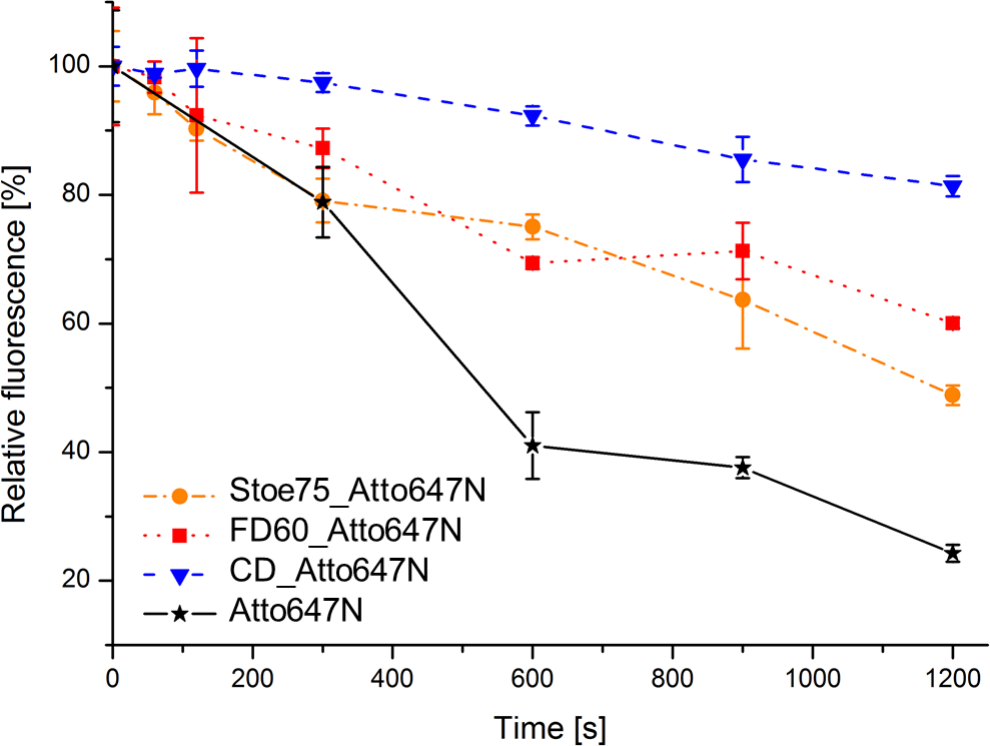
Overview of the photostability measurements of free Atto647N, Stoe75_Atto647N, FD60_Atto647N and CD_Atto647N. The samples were exposed to light from seven LEDs (λ_em_ = 594–650 nm) and the fluorescence intensity was measured over a period of 20 min.

In addition to high quantum yields and a high brightness, the fluorescence stability of the dye is of particular importance for STED imaging [59]. Especially, when this technique is used to address more complex tasks such as time-resolved or three-dimensional imaging. Therefore, the photostability of the synthesised dye-modified silica nanoparticles was confirmed by different photobleaching experiments. For this purpose, the three different nanoparticle suspensions were diluted to a particle concentration of 0.26 nM. A 40 nM solution of the free Atto647N–CS–APTES conjugate in MilliQ water was used as a reference to determine the influence of the embedding into the silica matrix. All sample solutions were exposed to light from seven commercially available LEDs (λ_em_ = 594–650 nm) and the fluorescence intensity was measured over a period of 20 min in order to compare their photobleaching. Even though the dye Atto647N is well known in literature for its high photostability, it showed a decrease of fluorescence intensity of around 75% after 20 min of continuously irradiation by the LEDs (Figure 5). However, the dyes that were embedded into the silica matrix show in each of the nanoparticle samples a higher photostability. The relative fluorescence of Stoe75_Atto647N and FD60_Atto647N dropped only to 48% and 61% of their starting intensity, respectively, after 20 min of continuous irradiation. The fluorescence intensity of CD_Atto647N fell only to about 81% of the initial value. We hypothesise that the differences in the decrease of fluorescence intensities can be explained by the varying thicknesses of the silica shells of the particles. Using the C-dots method, dye-doped silica nanoparticles with a dye-rich core and a pure silica shell are obtained [39]. In contrast, the two other methods lead to a significant distribution of the fluorescent dye in the particle matrix and consequently to a reduced shielding. To examine the hypothesis, an additional silica shell without dye molecules was applied on the FD45_Atto647N particles (FD45_Atto647N_NFSS). Next, the photostability of these particles and the fully dyed FD60_Atto647N was compared (Figure S21, Supporting Information File 1). As a result, a higher photostability can be achieved through an additional pure silica shell, since the fluorescence intensity of FD45_Atto647N_NFSS tends to be reduced slower compared to the fully dyed particles. This should be further increased by a thicker silica shell. A similar outcome was observed with separate samples of all nanoparticles that were only illuminated by day light (Figure S21, Supporting Information File 1). An extra pure silica shell provides protection from outside influences and makes the particles more stable against bleaching.

### Particle stability in cell culture medium

To investigate the behaviour of nanoparticles under experimentally relevant conditions with regard to in vitro or in vivo applications, the particle stability was tested in cell culture media, which contain high amounts of salts, amino acids and proteins. In aqueous media, electrostatic repulsion contributes to particle stabilisation. In presence of salts at neutral pH values, this type of stabilisation might be ineffective. In contrast, the formation of a protein corona at the interface between particle surface and medium might also influence the particle stability [60]. Thus, we tested the fluorescently labelled particles in MilliQ water and minimum essential medium (MEM) containing 20% of FBS (fetal bovine serum). Table 5 shows the amount of agglomerates 24 h after incubation of the particles in MilliQ water and MEM/FBS (20%) medium. For Atto647N-loaded nanoparticles no significant agglomeration was observed. However, Dy648-APTES particles showed a trend towards agglomeration with increasing size. The agglomerate content increased from 0.5% (40 nm) to 2.5% for 80 nm large particles. In addition, we tested a potential influence of the medium on the leaching of the Atto647N molecules. It demonstrated that the leaching of Atto647N was not negatively affected by the selected medium.

**Table 5:** Stability of Atto647N- and Dy-648-modified silica particles measured and calculated by dynamic light scattering. Particles were either diluted (1:100 v/v) with ultrapure H_2_O or MEM/FBS (20%) medium and incubated for 24 h before the mass-weighted agglomerate content was calculated. Numbers in brackets represent the size dispersity in percent.

dye	hydrodynamic diameter [nm]	agglomerate content [%]	
		
		H_2_O	Medium

FD25_APTES-Dy648	40 (30)	0	0.2
FD45_APTES-Dy648	60 (13)	1.7	1.4
FD60_APTES-Dy648	83 (10)	0	2.5
FD25_Atto647N	32 (20)	0.1	0.1
FD45_Atto647N	51 (13)	1.2	2
FD60_Atto647N	83 (14)	0	0.1

### Confocal and STED imaging of fluorescently labelled nanoparticles

**Figure 6:**
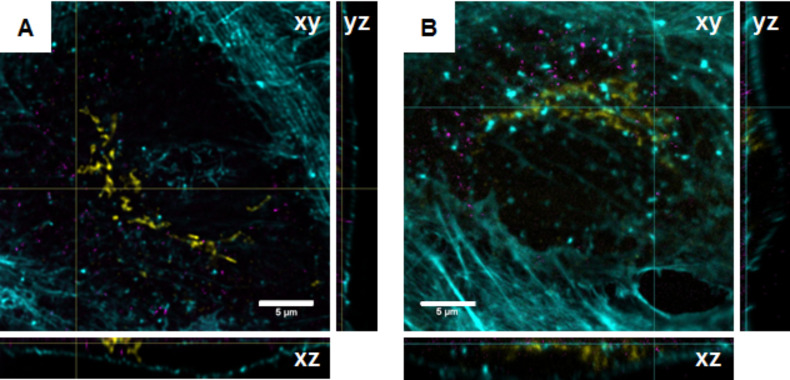
Confocal microscopic images of A549 cells exposed to 10 µg/mL FD25_Star635 (A) or FD25_Atto647N (B) for 24 h. Images represent the *x*–*y*, *x*–*z* and *y*–*z* sections derived from confocal *z*-stacks. The orthogonal projections indicate the intracellular location of the particles. The actin cytoskeleton is depicted in cyan, the cis-Golgi network in yellow and silica nanoparticles in magenta.

Finally, the potential use of the dye-modified silica particles as fluorescent probes in confocal and STED imaging was studied. For this investigation, A549 cells as model for alveolar epithelial type II cells were exposed to FD25_Atto647N and FD25_Star635 nanoparticles at a concentration of 10 µg SiO_2_ per mL. Confocal imaging revealed that the cells took up both particle types (Figure 6). The nanoparticles appeared to accumulate in the perinuclear region, but did not exclusively co-localise with the cis-Golgi network close to the nucleus, irrespective of the used label. Intensity profiles, derived from particle signals during STED imaging, revealed that a resolution of 85 and 88 nm (full width at half-maximum) was achieved (Figure S22, Supporting Information File 1), respectively. In comparison, a resolution of about 250 nm was determined by use of conventional confocal imaging (data not shown), representing a three-fold resolution enhancement.

## Conclusion

In this work, we report on the detailed synthesis and characterisation of NIR dye-labelled silica nanoparticles with sizes of 15 to 80 nm. Since most of the syntheses in this size-regime, which are based on the Stöber method or the microemulsion method, require harsh conditions and yield only particles with a high dispersity, we adapted the synthesis method described by Hartlen et al. [49]. We synthesised particles with modified cysteic acid–Atto647N dye conjugates incorporated into the silica matrix by multiple regrowth steps of 25 nm large silica nanoparticles. Dyes with different net electrical charges were used to study their influence on the particle synthesis. A neutral dye (Star635) and the negatively charged Dyomic dyes (Dy-647, Dy-648, Dy-649) could be directly incorporated into the silica matrix with an APTES linker. Although the particles modified with dye–cysteic acid conjugates show slightly better results, all particles were of spherical morphology and exhibited an excellent narrow size dispersity (below 10%). Variation of the dye concentration during the multiple synthesis steps resulted in no significant influence on the physical particle attributes. Further, the fluorescence characteristics, e.g., brightness and photostability of the probes are promising for their future application in high-resolution fluorescent imaging. Additionally, particle stability in biological media was tested by DLS after particle incubation in MEM/FBS (20%) medium for 24 h. All particles indicated only a low tendency to agglomerate, which makes them suitable for biological nanoparticle uptake experiments. Not only allows the presented synthesis method for the embedding of different kinds of fluorophores (proteins, quantum dots or metallic nanoclusters), but the method also provide the option to gain multicolour silica particles with or without Förster resonance energy transfer (FRET). These different approaches are currently under investigation.

## Experimental

### Materials

All syntheses and purification steps in aqueous solution were conducted in ultrapure water (18.2 MΩ·cm, MilliQ water purification system type ELIX 20, Millipore Corp., USA). L-arginine, cysteic acid monohydrate, tetraethoxysilane (TEOS), (3-aminopropyl)triethoxysilane (APTES), triethylamine (NEt_3_), *N*-hydroxysuccinimide (NHS), 1-ethyl-3-(3-dimethylamino-propyl)carbodiimide (EDC), cyclohexane, dimethyl sulfoxide (DMSO) and ethanol were purchased from Sigma-Aldrich (Taufkirchen, Germany) in the highest purity available and used as received without further purification. The fluorescent dyes Atto647N, Star635, Dy-647, Dy-648 and Dy-649 were purchased in the form of their reactive NHS-ester derivates from AttoTec (Siegen, Germany), Abberior (Göttingen, Germany) and Dyomics (Jena, Germany) and stored at −20 °C prior to use. All procedures, which involved the active dye–NHS esters, were performed under exclusion of moisture and light.

### Chemical syntheses of the dye–(cysteic acid)–APTES conjugates

**Dye–APTES conjugates (Star635-APTES, Dy647-APTES, Dy648-APTES or Dy649-APTES):** A similar approach was chosen for all dyes. In the following, the typical synthesis of Star635-APTES is described. 1 mg (1.01 µmol) of Star635–NHS ester was warmed to room temperature in a 2 mL vial. After dissolving the ester in 1 mL of water-free DMSO, 0.92 µL of APTES (3.94 µmol) was added and the mixture was reacted overnight at room temperature under constant shaking. The obtained dye–APTES conjugates were stored at −20 °C and used without further purification.

**Atto647N–cysteic acid–APTES conjugate (Atto647N-CS-APTES):** Atto647N–NHS ester (0.20 mg, 0.28 µmol) was warmed to room temperature in a 2 mL vial. After dissolving the dye in 200 µL of water-free DMSO, 47.40 µL of a 15 mM cysteic acid monohydrate solution (0.71 µmol) and an equal volume of 23.80 mM (1.13 µmol) NEt_3_ solution in DMSO were added and the mixture was kept under stirring at room temperature overnight. In a next step, 5 µL of NHS (stock solution: 10 mg/mL in DMSO) and 3.68 µL of EDC (stock solution: 11.36 µL/mL in DMSO) were added. Then the reaction mixture was stirred for 30 min at room temperature to allow for active ester formation, before 0.17 µL (0.72 µmol) of APTES was added. Further stirring overnight yielded the silanol-reactive linker, which was used without further purification. A similar approach was chosen for the other dyes (Star635-CS-APTES and Dy-CS-APTES). For their synthesis 0.23 mg (0.28 µmol) Star635 or 0.25 mg (0.28 µmol) Dy648 were used, respectively.

### Chemical syntheses of dye-modified silica nanoparticles

**Synthesis of 15 nm fully dyed Atto 647N silica nanoparticles (FD15_Atto647N):** 91 mg (0.53 mmol) L-arginine was dissolved in 69 mL of water. Next 4.5 mL of cyclohexane was added to the filtered L-arginine solution and the biphasic mixture was heated to 40 °C under stirring. Particle formation was induced by the addition of 5.5 mL (28.1 mmol) TEOS. After 30 min, 100 µL (0.1 mmol) of Atto647N-CS-APTES linker solution was added. Further heating for 20 h yielded small silica particles of *d* = 15 nm with incorporated Atto647N dye.

**Synthesis of 25 nm dyed Atto647N silica nanoparticles (FD25_Atto647N):** The synthesis of 25 nm large Atto647N dyed silica particles was realised analogous to the procedure described above. Only the temperature of the heating process was changed to 60 °C.

**Synthesis of fluorescent silica core–shell particles using multiple regrowth steps (FD45; FD60 and FD80):** Here, the synthesis of FD45_Atto647N is described exemplarily for the multiple regrowth steps. 3.52 mL (15.88 mmol) of TEOS was added at 60 °C under stirring to a suspension containing 10 mL of silica core solution (FD25_Atto647N), 14 mg (0.09 mmol) L-arginine, 36 mL of water and 4.5 mL of cyclohexane. The biphasic system was kept at 60 °C for 30 min before 100 µL (0.1 mmol) of Atto647N-CS-APTES conjugate was added and the resulted mixture was further stirred for 20 h at 60 °C. The obtained particles indicated an increase of the particle size by around 20 nm. The shell thickness can be reduced by stopping the reaction after TEOS addition at *t* = 12 h or 6 h. Multiple fluorescent shells (FD60_Atto647N or FD80_Atto647N) can be achieved by repeating the described procedure. In a similar way Dy-648-modified particles with different sizes could be prepared using the Dy648–APTES conjugate.

**Synthesis of fluorescent 15 nm and 60 nm large Stöber particles dyed with Atto647N-CS-APTES conjugate (Stoe25_Atto647N and Stoe75_Atto647N):** Atto647N-modified silica nanoparticles using the Stöber approach were synthesised by adding 54 µL (0.06 mmol) of Atto647N–CS–APTES conjugate into the water/ethanol/ammonia mixture (0.41 mL/23.57 mL/0.50 mL) directly before the corresponding amount of TEOS. The reaction mixture was stirred for 2 d at room temperature. To gain larger dyed silica nanoparticles (*d* = 60 nm) the same amount of dye was added to the changed ratio of TEOS (1.05 mL, 4.7 mmol) and the water/ethanol/ammonia mixture (0.41 mL/22.55 mL/1.04 mL).

**Synthesis of Atto 647N-labelled C-dots (CD_Atto647N):** The dye-labelled C-dots were synthesized by a small variation of the method described above for the non-labelled nanoparticles. For the typical nanoparticle synthesis of 25 mL scale, 0.385 mL of MilliQ water, 21.75 mL of ethanol and 2.5 mL of ammonia were mixed and stirred for 10 min at room temperature. Next, 0.28 mL (1.26 mmol) of TEOS and 0.09 mL (0.09 mmol) of Atto647N–CS–APTES were added and the reaction mixture was stirred for another 12 h to form the particle cores. Addition of further TEOS aliquots (0.105 M) to the core solution, at a rate of 1 µL per mL of reaction volume every 15 minutes, produced a pure silica shell. After stirring of the reaction mixture for additionally 8 h, 15 nm large Atto647N dye nanoparticles were obtained.

### Purification

After their synthesis, all the particles were purified by dialysis against water (4 L, water exchange after 30 min, 1 h, 2 h, 12 h) with dialysis membrane (Nadir, regenerated cellulose membrane, molecular weight cut-off: 10–20 kDa) from Carl Roth (Karlsruhe, Germany), followed by filtration using a sterile 0.22 µm cellulose acetate membrane. After purification 25 nm silica nanoparticle suspensions containing between 800 and 1200 mg SiO_2_·L^−1^ were obtained.

Smaller silica cores could be prepared by control of the reaction temperature. Larger silica cores could be obtained by several regrowth steps using lower L-arginine concentrations.

See Supporting Information File 1 for the synthesis of pure silica nanoparticles and different dye embedded nanoparticles.

### Characterisation

To determine the particle diameters, the morphology and the agglomeration state, the samples were analysed by dynamic light scattering measurements (DLS), scanning electron microscopy (SEM) and transmission electron microscopy (TEM).

**Dynamic light scattering:** DLS measurements were performed with a StabiSizer Nano 250 from Particle Metrix (Meerbusch, Germany) or the Nanosizer ZSP from Malvern Instruments (Herrenberg, Germany). For the DLS measurements the particle dispersions were diluted 1:10 in MilliQ water. Measurements were performed at room temperature. Each sample was measured three times with at least three runs (3 min/run). The mean hydrodynamic diameter was determined by using the cumulants analysis and a size distribution using a regularization scheme by volume.

**Electron microscopy:** SEM images were taken using a FEI Quanta 400 ESEM FEG system (FEI Company, Netherlands). For this purpose undiluted samples were dried on a silicon wafer and images were taken under high vacuum using acceleration voltages of up to 20 kV. TEM measurements were carried out using a Philips CM200 FEG (FEI Company, Netherlands). The samples were prepared by immersion of a 200-mesh carbon-coated copper grid into the nanoparticle suspension. The average primary particle size and the particle size distribution were determined by analysing SEM and TEM images using the software ImageJ (Version: 1.45a; http://rsbweb.nih.gov/ij/). In brief, after background subtraction and adjustment of brightness and contrast the SEM or TEM images were converted to 8-bit binary images. Particle size diameter and shape were counted automatically.

**Zeta-potential measurements:** The ζ-potential of the nanoparticles was determined using a Nanosizer ZSP from Malvern Instruments (Herrenberg, Germany) in water at 150 V using 1·10^−3^ M KCl as background electrolyte. Each sample underwent three series of measurements (with each series comprising 40 runs).

**Nanoparticle concentration:** The nanoparticle number concentrations were calculated based on the determined particle diameter from the TEM measurements and the SiO_2_ content, which was obtained by ICP-OES (Ultima 2, Horiba Jobin Yvon, Japan).

**Absorption and fluorescence measurements:** All optical measurements were performed at room temperature under ambient conditions. The UV–vis spectra of diluted and undiluted solutions were recorded with a Cary 5000 spectrophotometer (Varian Inc., Darmstadt, Germany) in the range from 300 to 800 nm. Fluorescence spectra were obtained with a spectrofluorometer Spex FluoroMax-3 from HORIBA Jobin Yvon (Oberursel, Germany) using a xenon lamp and different excitation wavelengths. The used nanoparticle suspensions were diluted (1:10 to 1:1000) in MilliQ water. Samples were placed in a cuvette (1 cm path length) for both fluorescence and UV–vis measurements. The brightness of the particles was quantified in comparison to the free dye Atto647N by standard absorption matching between free dye and particle solutions plus a subsequent emission investigation of the matched solutions. The quantum yield values were determined from the equation:


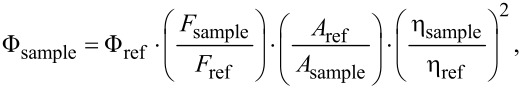


where *F*, *A* and η are the measured fluorescence, the measured absorbance and the refractive index of the solvent, respectively [56]. When performing this type of comparative measurement, all the measurement parameters were identical for the reference sample and the sample to be tested.

### Exposure of cells to NPs and preparation of samples for microscopy

Nanoparticle uptake experiments were performed using A549 cells (DSMZ-number: ACC 107) as model for alveolar epithelial type II cells. Cells were cultivated in Dulbecco’s modified Eagle medium (DMEM, Gibco, Life Technologies, USA) supplemented with 10% fetal bovine serum (FBS, PAN biotech, Germany) in a humidified incubator (37 °C, 9% CO_2_). The cells were seeded on cover slips with a density of 1·10^5^ cells per cm^2^ and allowed to attach for 24 h. After 24 h of incubation in presence of the nanoparticles, the medium was removed and the cells were washed two times with Dulbecco's phosphate-buffered saline (DPBS, Gibco, USA), fixed with paraformaldehyde (4% in DPBS) for 30 min at room temperature and permeabilised with Triton X-100 (0.2% in DPBS) for 15 min at room temperature. Unspecific binding sites were blocked with BSA (5% in DPBS) for 30 min at room temperature. Phalloidin-488 (A12379, 300 U, Thermo Fisher Scientific; 1 h, 1:40 in PBS containing 1% BSA) and GM130 primary antibody (610822, 250 µg/mL, BD Biosciences; 1 h, 25 µg/mL in PBS containing 1% BSA) with Alexa546 goat anti mouse antibody (A 11030, Thermo Fisher Scientific; 1 h, 1:500 in PBS containing 1% BSA) were used to label the actin cytoskeleton and the cis-Golgi apparatus, respectively. Cells were mounted on glass slides with Mowiol/DABCO (Sigma-Aldrich, Taufkirchen, Germany) 1 h, 1 µL in 500 µL 1% BSA/PBS.

### STED Imaging

STED imaging was performed using a Leica TCS-SP5 STED (Leica Microsystems, Mannheim, Germany) microscope equipped with a Leica HCX PLAN APO 100×/1.4 oil immersion objective The 488 nm laser line of an argon laser and a 561 nm DPSS laser were used for excitation of Alexa Fluor 488 and Alexa Fluor 546, respectively. The labelled NPs were imaged in STED mode using a pulsed 635 nm laser diode (PicoQuant, Berlin, Germany) for excitation and an infrared laser (MaiTai, Spectra Physics, Santa Clara, United States) running at 750 nm for STED depletion.

## Supporting Information

Additional experimental data and images are found in Supporting Information File 1.

File 1 Additional experimental data.
