# Supplementary material for: Bright fluorescent silica-nanoparticle probes for high-resolution STED and confocal microscopy
Source: Beilstein J Nanotechnol. 2017 Jun 21;8:1283–96. doi: 10.3762/bjnano.8.130 (PMC5496580; doi:10.3762/bjnano.8.130)
Supplement: File 1 — Additional experimental data. [file Beilstein_J_Nanotechnol-08-1283-s001.pdf]

Supporting Information

for

Bright fluorescent silica-nanoparticle probes for high-resolution STED and confocal microscopy

Isabella Tavernaro*[1], Christian Cavelius[2], Henrike Peuschel[1] and Annette Kraegeloh[1]

Address: [1]INM - Leibniz Institute for New Materials, Nano Cell Interactions Group, Campus D2 2, D-66123 Saarbrücken, Germany and [2]present address: nanoSaar Lab GmbH, Comotorstr. 2, D-66802 Überherrn, Germany

Email: Isabella Tavernaro* - isabella.tavernaro@leibniz-inm.de

* Corresponding author

Additional experimental data

Contents

1. Synthesis of pure silica nanoparticles
2. Synthesis of dyed silica nanoparticles
3. Characterisation of non-dyed silica nanoparticles
4. Characterisation of dyed silica nanoparticles
5. Comparison of the different synthesis methods
6. Absorption and fluorescent measurements
7. Cell tests
8. References

1. Synthesis of pure silica nanoparticles

Synthesis of pure silica nanoparticles (Me_series). Synthesis of pure silica nanoparticles using L-arginine as a base catalyst was conducted as described in the literature [1]. In a typical experiment, 91 mg (0.52 mmol) L-arginine was dissolved in 69 mL of water. After addition of 4.5 mL of cyclohexane, the biphasic mixture was heated to 60 °C under stirring. Particle formation was induced by addition of 5.5 mL (28.1 mmol) TEOS and further heating for 20 h. Then the organic cyclohexane phase was separated from the aqueous particle suspensions.

Synthesis of pure silica nanoparticles by the Stöber method (Stoe15 and Stoe60). Undoped silica nanoparticles were synthesised by the Stöber method [2]. To obtain small silica nanoparticles with a mean particle size of 15 nm, 0.41 mL of water, 23.57 mL of ethanol and 0.50 mL of ammonia (25%) were mixed. In a next step 0.554 mL (2.5 mmol) of TEOS was added and the reaction mixture was stirred for 2 d at room temperature. For larger silica particles with a mean diameter of 60 nm, the precursor ratio was changed to 0.41 mL of water, 22.55 mL of ethanol, 1.04 mL of ammonia and 1.05 mL (4.7 mmol) of TEOS.

Synthesis of pure silica nanoparticles using the C-dot method (CD). Pure C-dots were synthesised following the method described by Herz *et al*. [3]. 0.28 mL (1.3 mmol) of TEOS was added to a mixture of 21.75 mL ethanol, 0.39 mL of water, 2.5 mL of ammonia (2.0 M in ethanol). Then the reaction mixture was stirred for 12 h at room temperature to obtain the particle cores. Addition of further TEOS aliquots to the core solution and stirring of the reaction mixture for additional 8 h formed a pure

silica shell around the cores. To avoid secondary nucleation 0.4 mL (1.8 mmol) of TEOS was added stepwise over a maximum period of 300 min (20 µL / 15 min).

2. Synthesis of dyed silica nanoparticles

Synthesis of 25 nm large, dyed Star635 silica nanoparticles (FD25_Star635). To determine the influence of the dye concentration on the particle growth, Star635 embedded silica particles with two different dye concentrations were synthesised. Due to this 91 mg (0.53 mmol) of L-arginine was dissolved in 69 mL of water and filtered through a 0.22 µm cellulose acetate membrane. To the L-arginine solution 4.5 mL of cyclohexane was added, the biphasic mixture was heated to 60 °C under stirring and the particle formation was induced by addition of 5.5 mL (28.1 mmol) TEOS. After 30 min, 132.15 µL (0.11 mmol) or 264.30 µL (0.22 mmol) respectively of Star635-CS-APTES linker solution was added. Further heating for 20 h yielded small monodisperse silica nanoparticles of 25 nm diameter with Star635 incorporated into the silica matrix.

Syntheses of Star635 or *Dyomics* dyes embedded silica nanoparticles using the dye-APTES conjugates. Similar to the synthesis of **FD25_Star635**, embedding of **Star635-APTES** was performed by dissolving 91 mg (0.53 mmol) of L-arginine in 69 mL of water, followed by addition of 4.5 mL cyclohexane. The biphasic mixture was heated to 60 °C under stirring. Particle formation was induced by addition of 5.5 mL (28.1 mmol) TEOS. After 30 min, 28 µL (0.03 mmol) of **Star635-APTES** linker solution was added. Further heating for 20 h yielded small silica particles with a

diameter of 33 nm. **FD_APTES_Dy647**, **FD_APTES_Dy648** and **FD_APTES_DY649** were synthesised in a similar way.

Synthesis of Atto647N-dyed nanoparticles with an additional non fluorescent silica shell (FD45_Atto647N_NFSS). Applying an additional non-fluorescent silica shell onto the dye doped silica nanoparticles was enabled through following the procedure of the regrowth steps without any addition of the dye. For this purpose 14 mg (0.08 mmol) L-arginine was dissolved in 36 mL of water followed by addition of 10 mL of **FD45_Atto647N** particle suspension. Next 4.5 mL of cyclohexane was added and the biphasic mixture was heated to 60 °C under stirring. Particle formation was induced by addition of 5.5 mL (28.1 mmol) TEOS. Further heating for 20 h yielded 60 nm large silica particles with Atto647N dye incorporated into the dye matrix and a non-fluorescent outer shell.

3. Characterisation of non-dyed silica nanoparticles

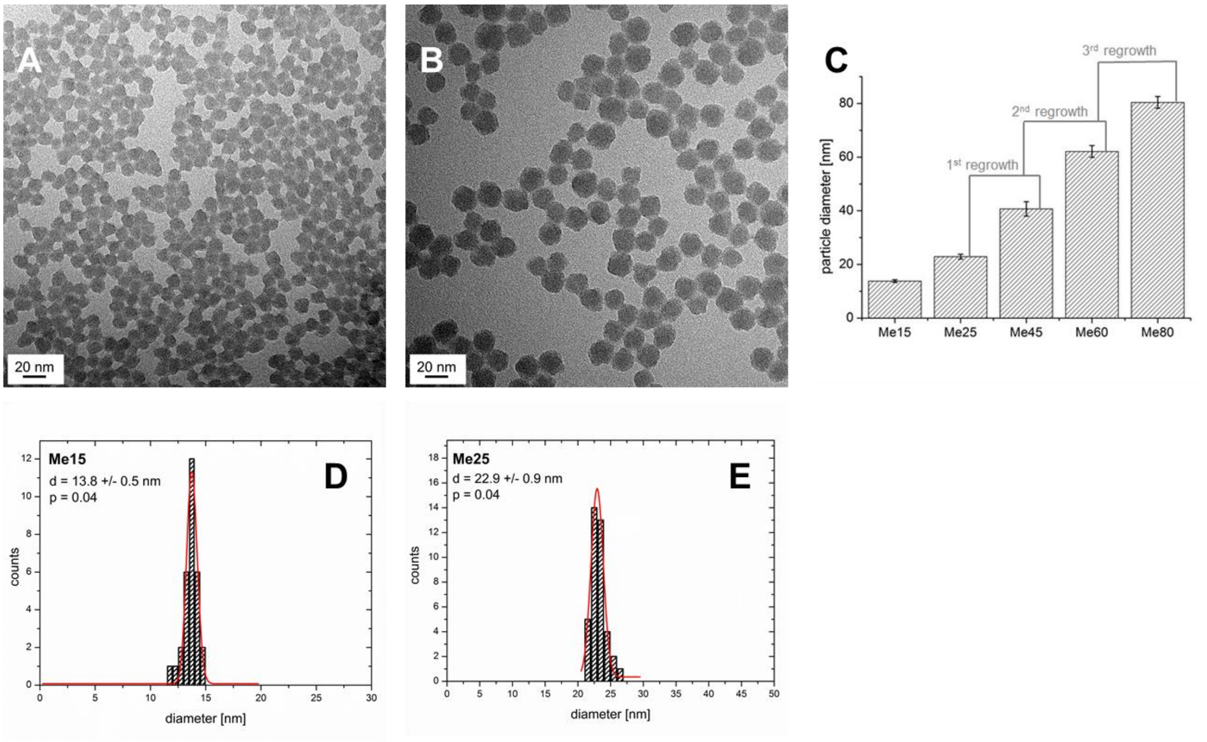

Figure S1: TEM images and histograms of synthesised silica nanoparticles without embedded dyes. TEM images of pure silica nanoparticles with a mean particle diameter of 14 nm (A) and 23 nm (B). C: The scheme shows the use of Me25 as a starting point for the syntheses of larger nanoparticles using multiple regrowth steps. Histograms derived from analysis of TEM images are indicated in D (Me15) and E (Me25).

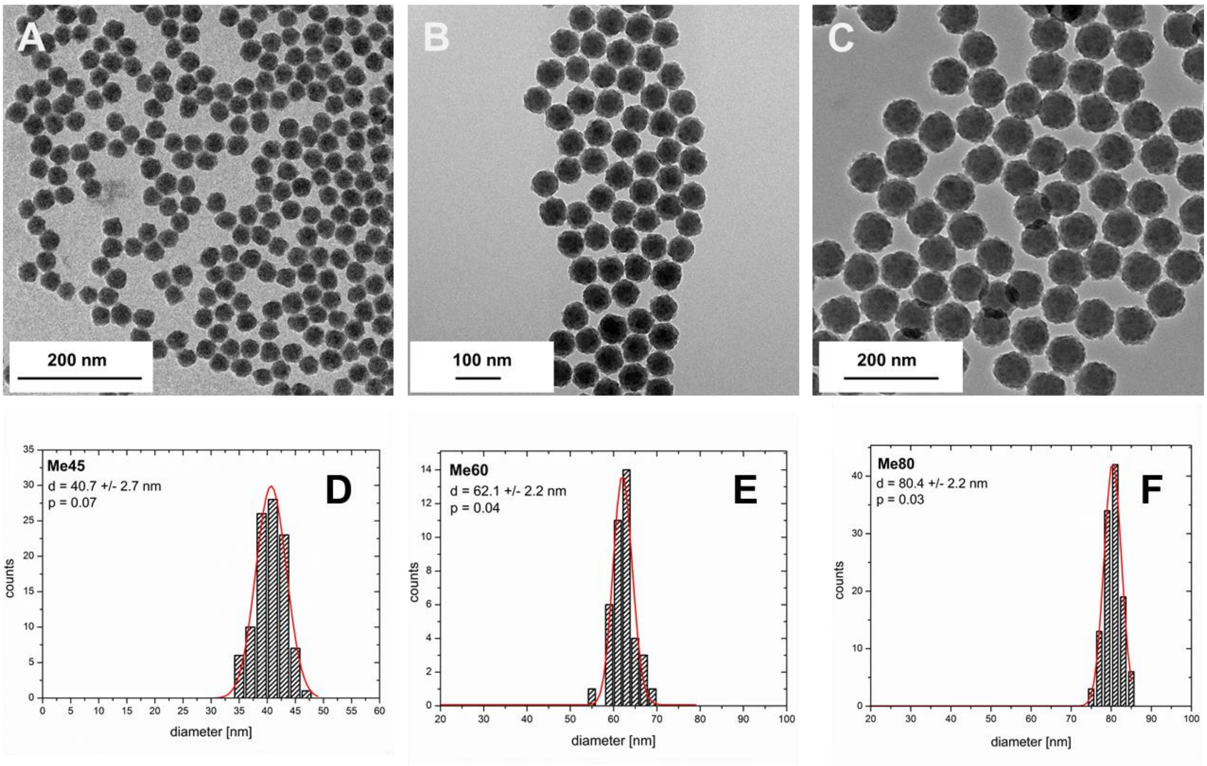

Figure S2: TEM images and histograms of pure silica nanoparticles without embedded dyes obtained by the multistep synthesis procedure. A: TEM image of pure silica nanoparticles with a mean particle diameter of 41 nm; B: TEM image of pure silica nanoparticles (Me60) obtained after the second regrowth step of Me25; C: TEM image of 80 nm large silica nanoparticles (Me80), which were obtained after the third regrowth step of Me25; D-F indicate the associated histograms.

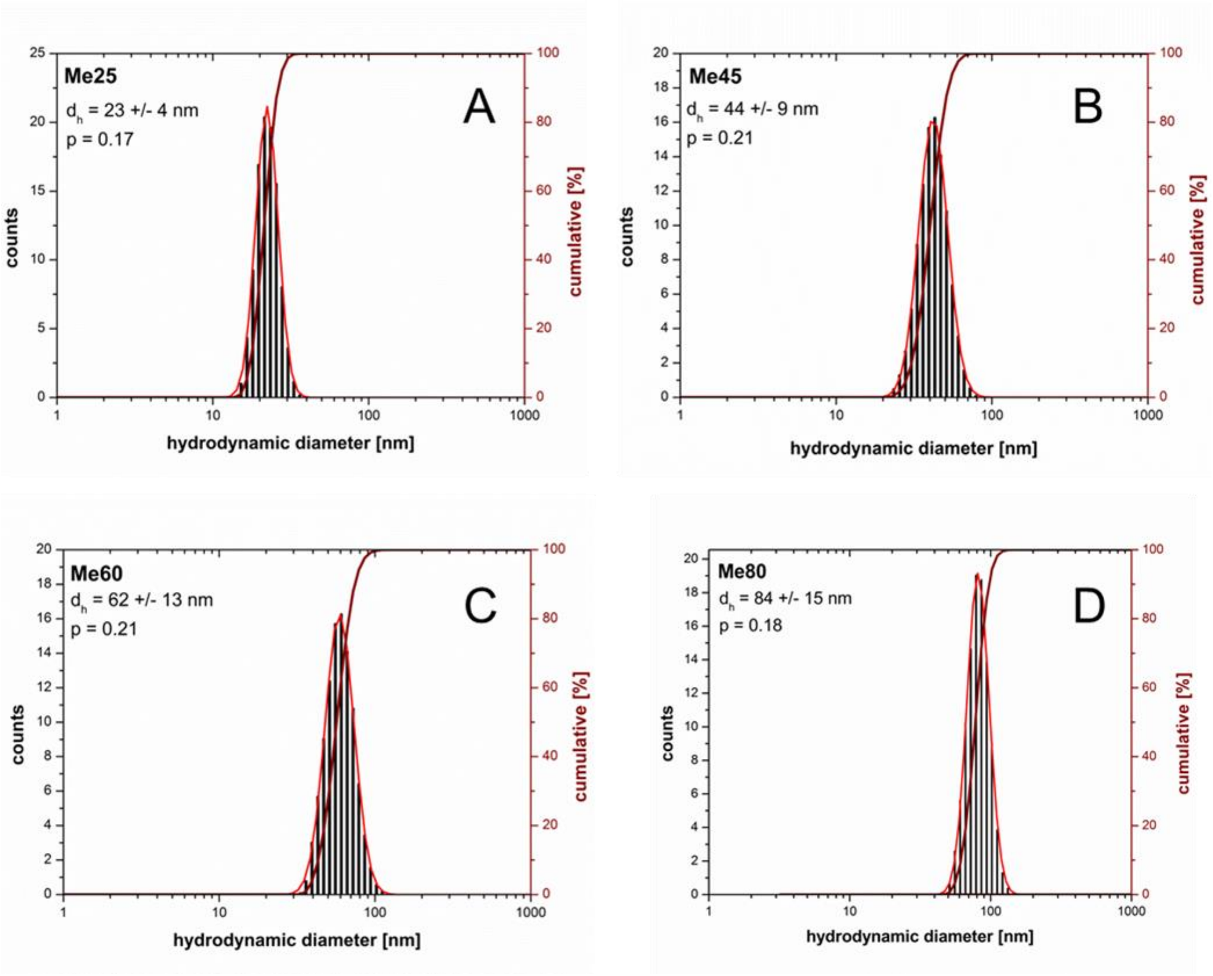

Figure S3: Results of the dynamic light scattering measurements of the non-dyed silica nanoparticles. A: results of Me25; B: results of Me45; C: results of Me60 and D: results of Me80. Determination of the hydrodynamic diameter (d_h) and dispersity (p) were performed using the volume distribution calculated from the autocorrelation function.

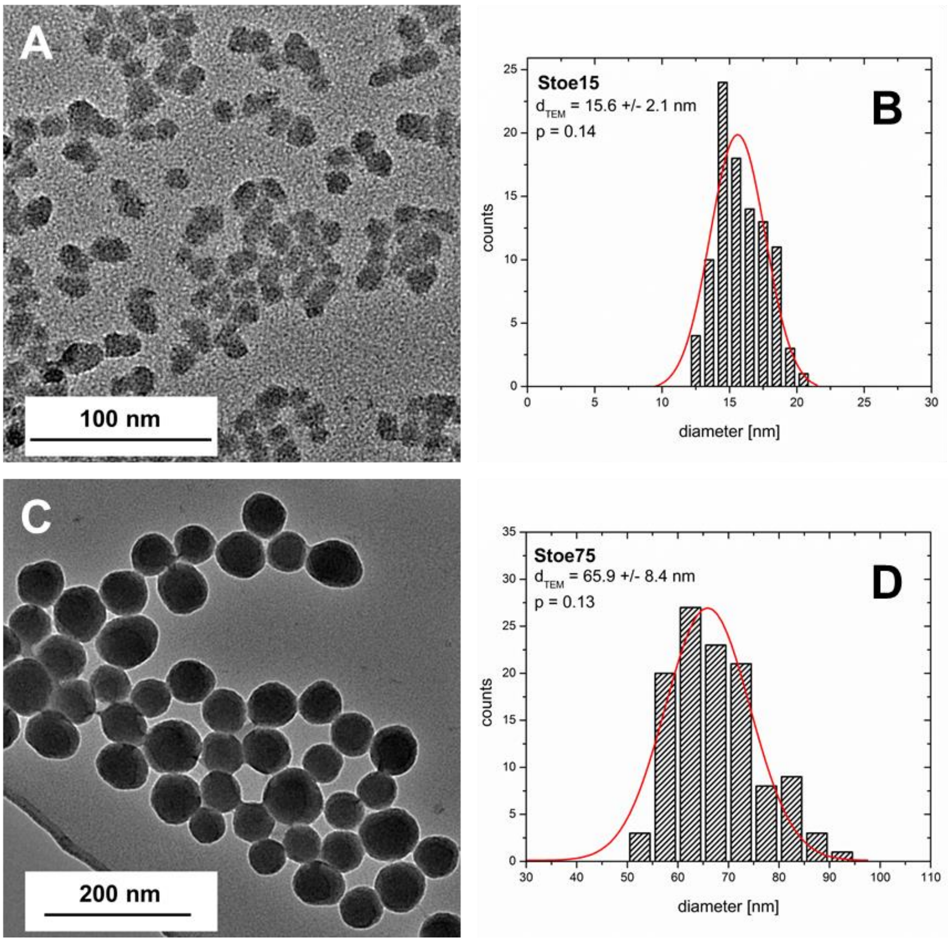

Figure S**4:** TEM images of the non-dyed silica nanoparticles, which were prepared by the literature known Stöber approach [2] (A and C). B and D indicate the associated histograms derived from the TEM images.

4. Characterisation of dye-doped silica nanoparticles

4.1 Characterisation of Atto647N-doped silica nanoparticles

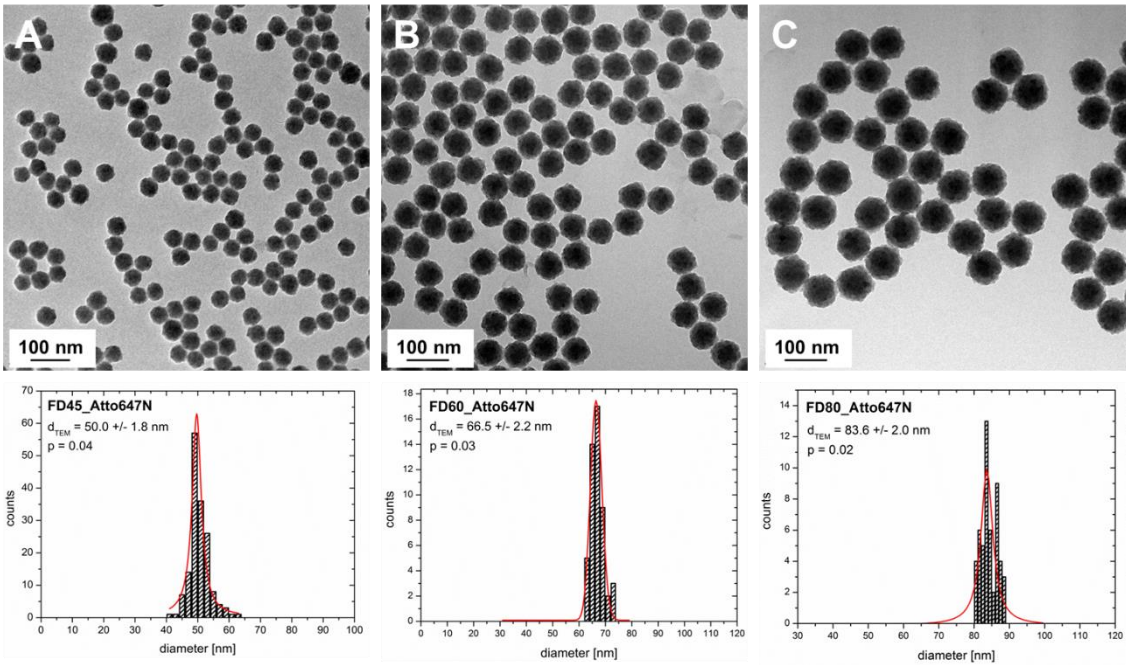

Figure S5: TEM images and histograms of FD45_Atto647N (A), FD60_Atto647N (B) and FD80_Atto647N (C).

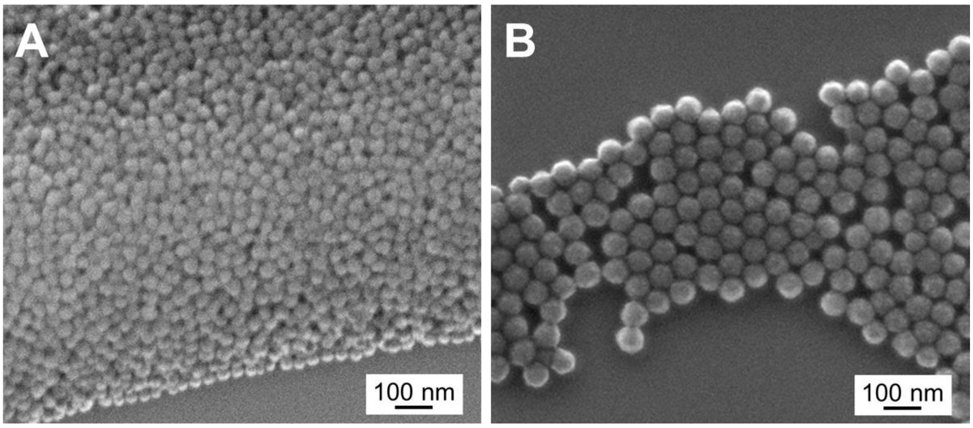

Figure S6: SEM images of FD25_Atto647N (A) and FD80_Atto647N (B).

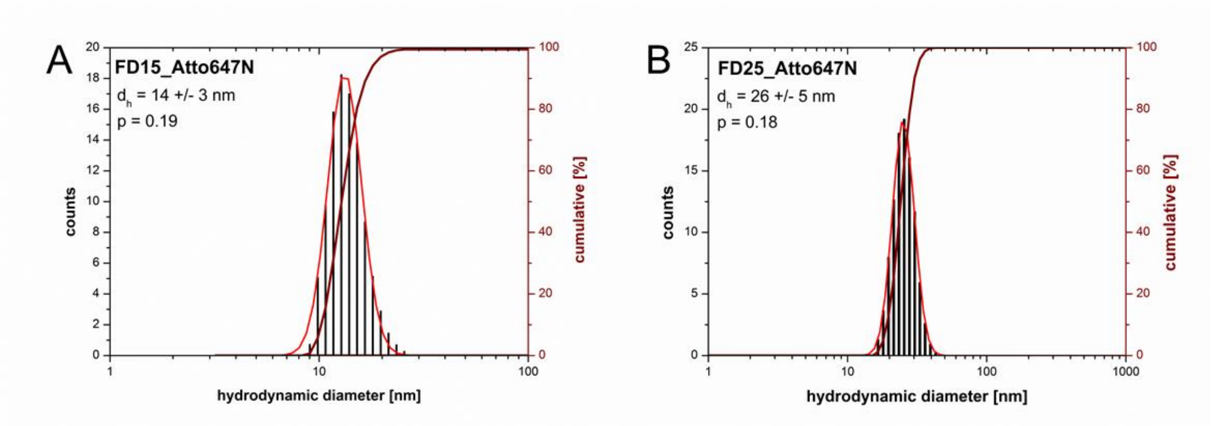

Figure S7: Results of DLS measurements of FD15_Atto647N (A) and FD25_Atto647N. Determination of the hydrodynamic diameter (d_h) and dispersity (p) were performed using the volume distribution calculated from the autocorrelation function.

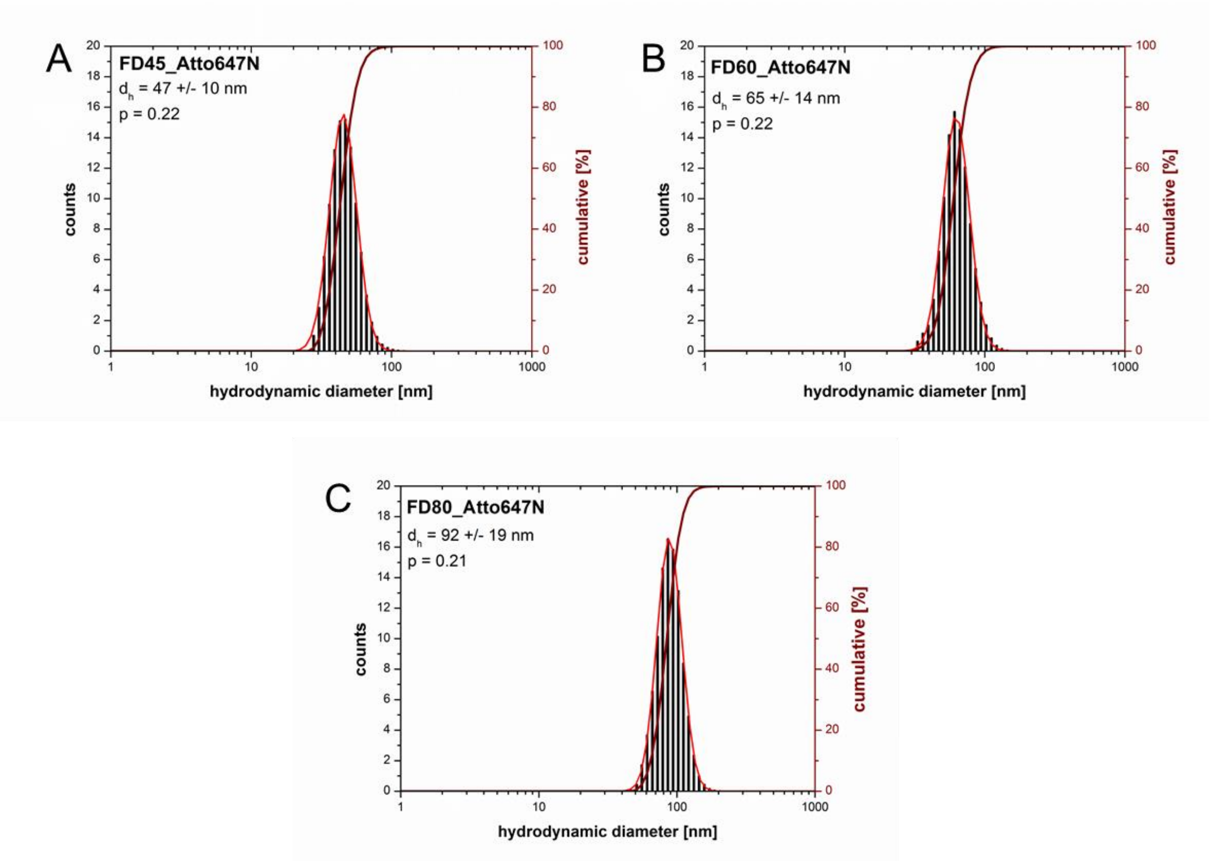

Figure S8: Results of DLS measurements of FD45_Atto647N (A), FD60_Atto647N (B) and FD80_Atto647N (C). Determination of the hydrodynamic diameter (d_h) and dispersity (p) were performed using the volume distribution calculated from the autocorrelation function.

4.2 Synthesis of silica nanoparticles embedded with other dyes

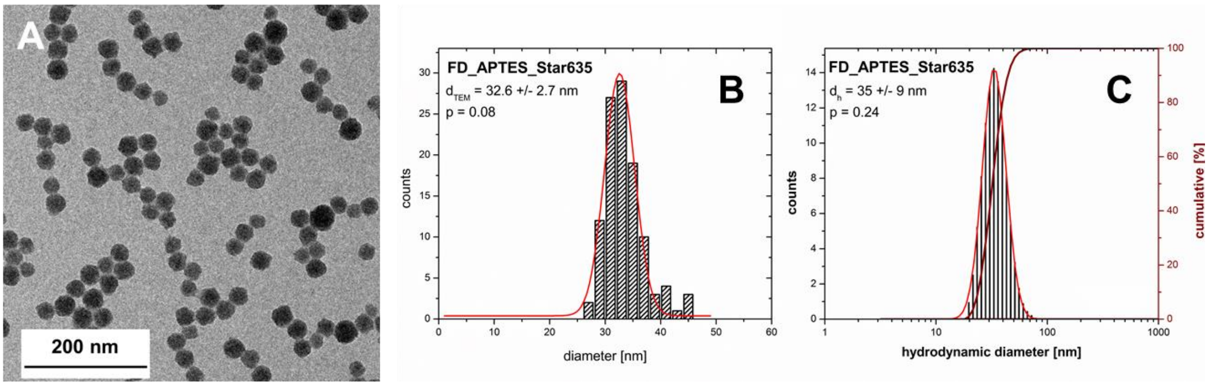

Figure S9: Analysis of physical particle attributes of FD_APTES_Star635: TEM image (A); histogram of FD_APTES_Star635 (B), derived from the TEM image analysis, and the results of DLS measurements (C).

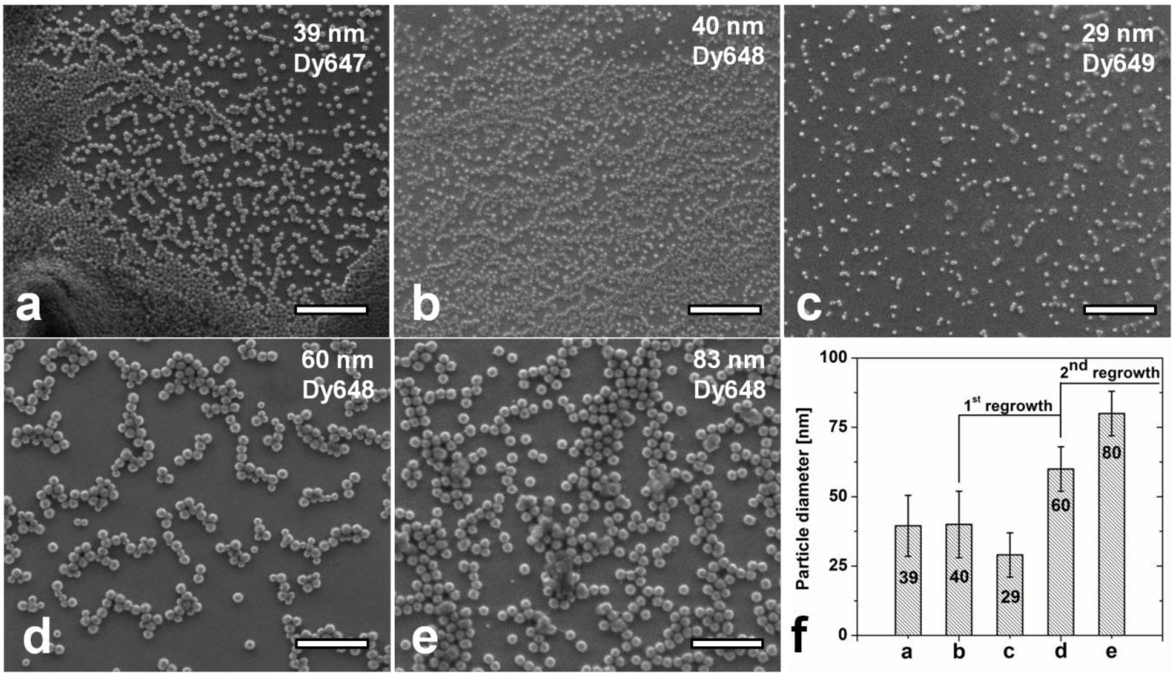

Figure S10: SEM images of (a) **FD_APTES-Dy647**: d_{SEM} = 39 ± 11 nm (28%); (b) **FD_APTES-Dy648**: d_{SEM} = 40 ± 12 nm (30%); and (c) **FD_APTES-Dy649**: d_{SEM} = 29 ± 8 nm (28%) silica particles obtained after covalent incorporation of the dye-APTES conjugates Dy647-APTES, Dy648-APTES and Dy649-APTES. Size and dispersity were similar for Dy-647 and Dy-648 dyes which carry one or two sulphonate groups, respectively. Smaller particles were obtained by introduction of Dy649 dye. A first regrowth step of (b) yielded particles with a mean diameter of d_{SEM} = 60 ± 8 nm (13%) (d) that could be regrown to 80 ± 8 nm (10%) large particles (e). The graph (f) shows the mean particle diameter and size deviation obtained from SEM image analysis and logNormal fitting of the histograms. All scale bars are 300 nm.

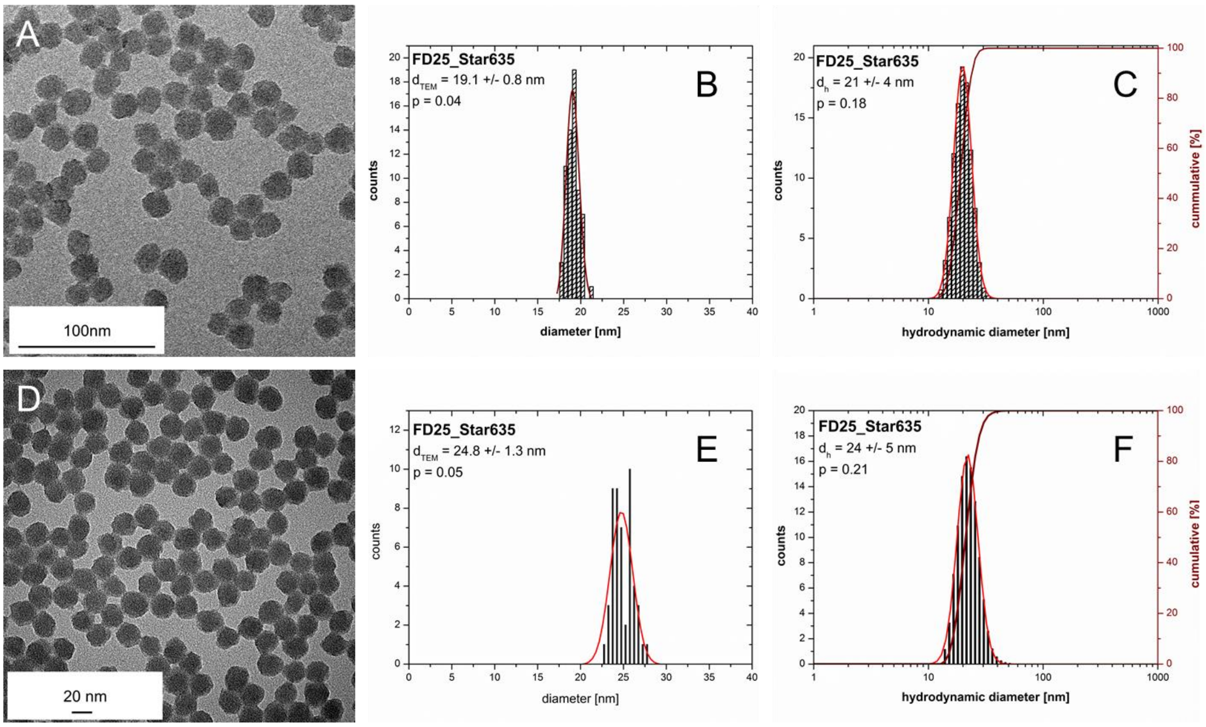

Figure S11: TEM images and DLS results of the synthesised silica nanoparticles dyed with different concentrations of Star635-CS-APTES conjugates. TEM image (A), histogram of TEM (B) and results of the DLS measurements (C) of FD25_Star635, using a dye concentration of 56.4 µL during the particle synthesis. TEM image (D), histogram of TEM (E) and results of the DLS measurements (F) using a higher concentration (112.8 µL) during the particle synthesis.

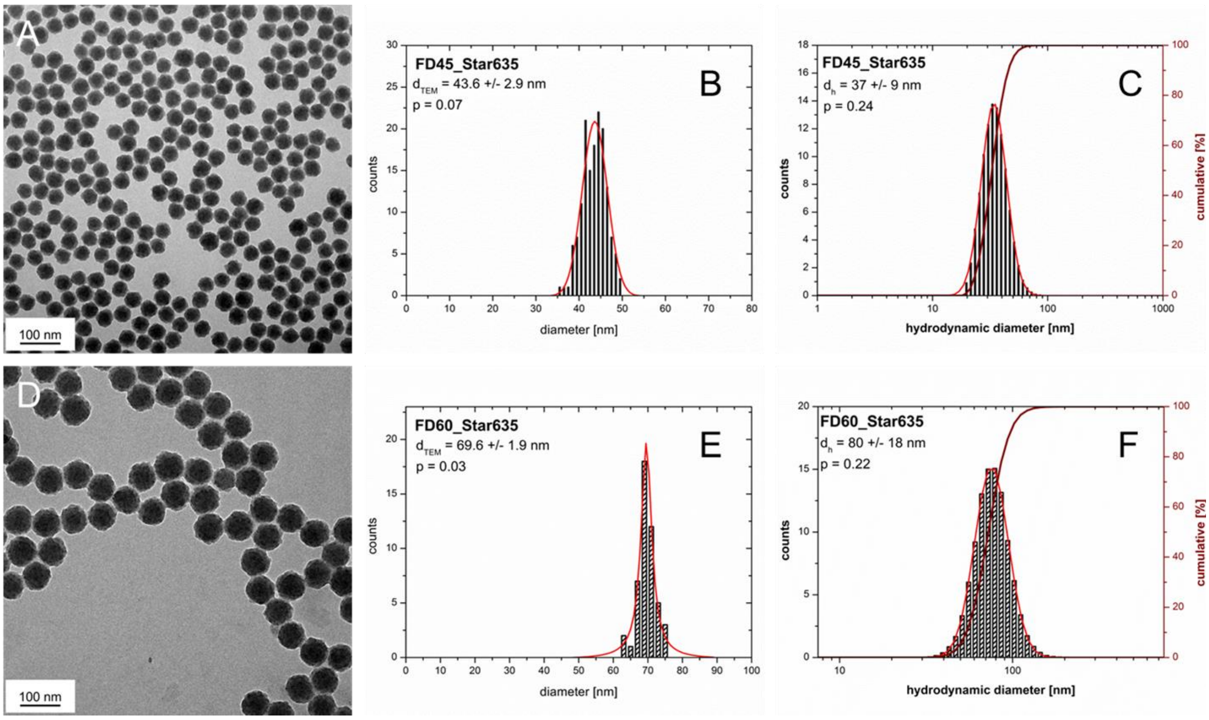

Figure S12: TEM images and DLS results of the obtained nanoparticles after the first and second regrowth step, using a twice as high dye concentration (112.8 µL, 0.09 mmol) of Star635_CS_APTES during the particle synthesis. TEM image (A), histogram of the TEM analysis (B) and results of the DLS measurements (C) of FD45_Star635. TEM image (D), histogram of the TEM analysis (E) and results of the DLS measurements (F) of FD60_Star635.

5. Comparison of different synthesis methods

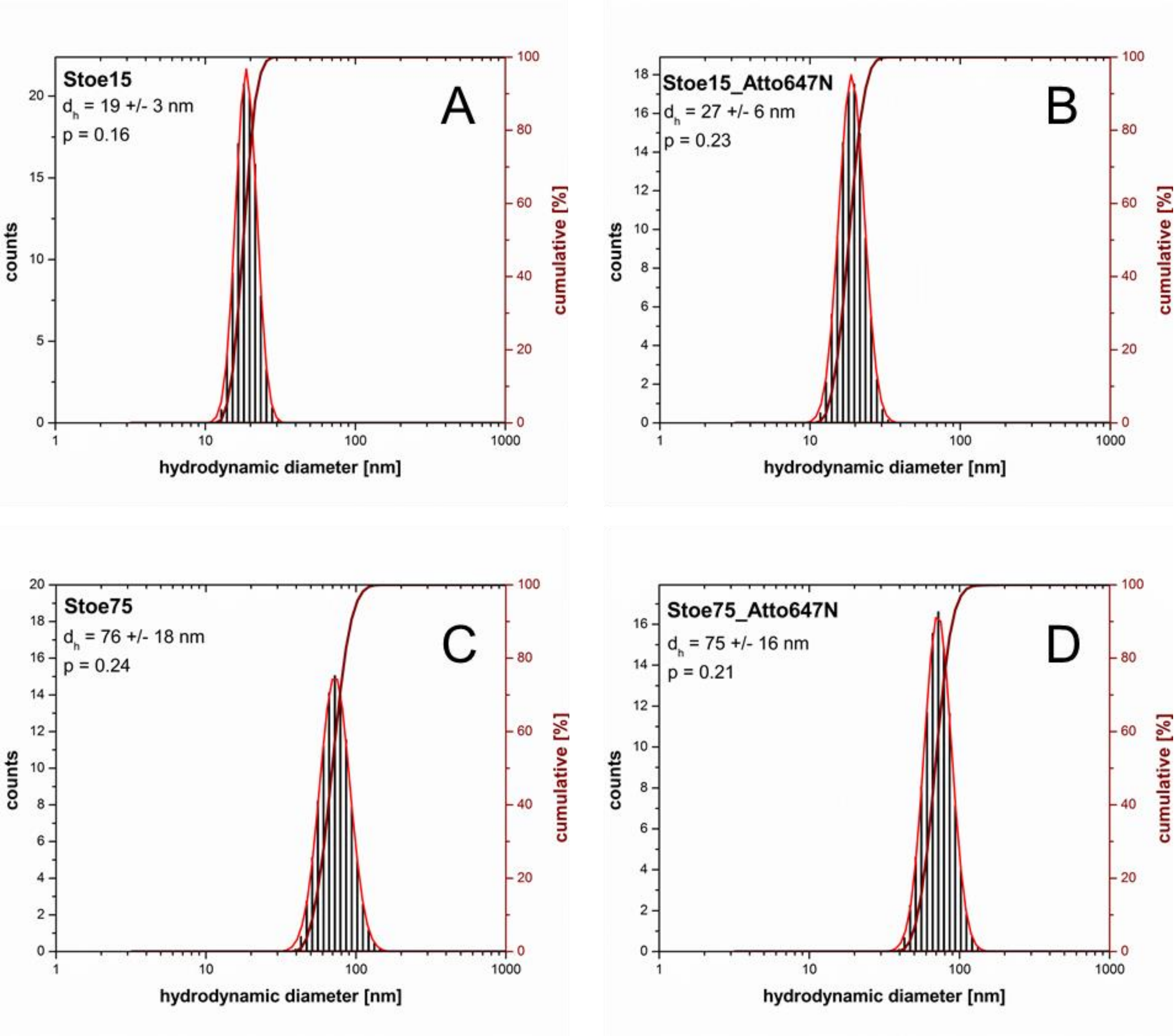

Figure S13: Results of the DLS measurements of silica nanoparticles obtained by the synthesis with the Stöber method.

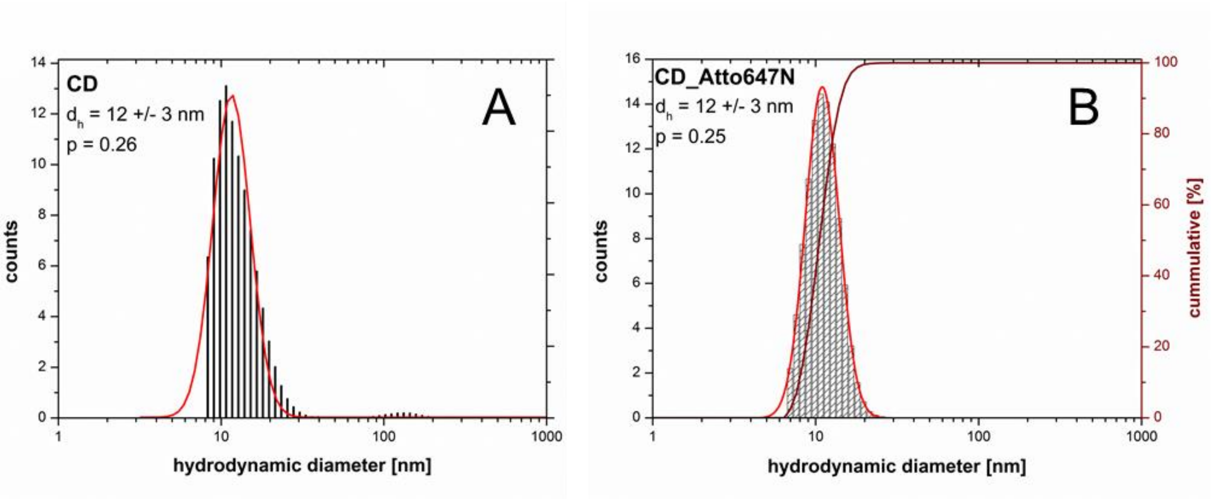

Figure S14: Results of the DLS measurements of silica nanoparticles synthesised with the C-dots method.

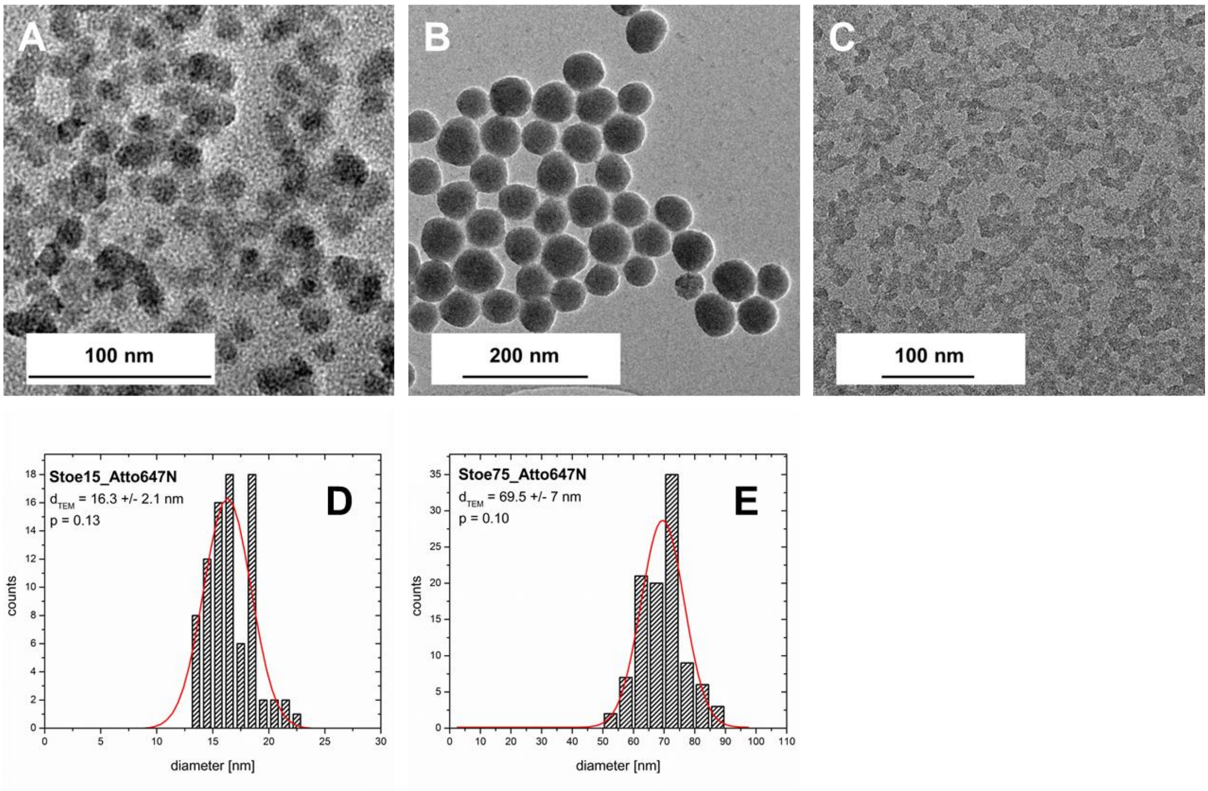

Figure S15: TEM images of the Atto647N-dyed silica nanoparticles, which were synthesized by the literature known Stöber approach (A and B) or the C-Dots method (C); C: CD_Atto647N with a mean particle size of d_{TEM} = 11.5 ± 0.7 nm. D and E indicate the histograms of the dyed silica nanoparticles derived from the associated TEM images (A and B).

6. Absorption and fluorescence measurements

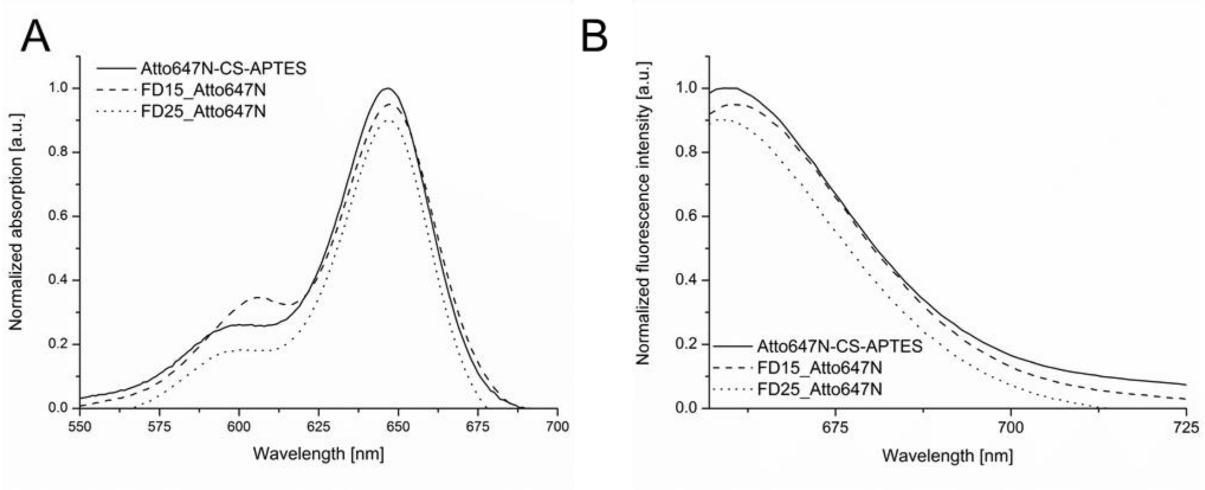

Figure S16: Normalized absorption (left) and emission (right) spectra of free Atto647N-CS-APTES and Atto647N dye-doped silica nanoparticles with different diameters (FD15_Atto647N and FD25_Atto647N). All absorption spectra indicate two maxima at 600 and 647 nm, whereas the emission spectra show one maximum at 660 nm.

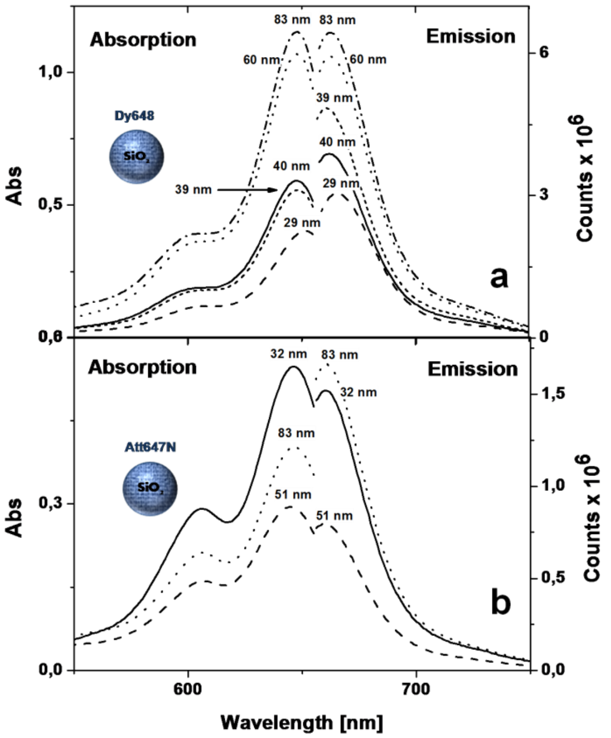

Figure S17: UV–vis (left axis) and fluorescence (right axis) spectra of silica nanoparticles obtained after *in situ* incorporation of modified Dy648-APTES conjugate (a) and Atto647N-CS-APTES conjugate (b) into the matrix material. Both dyes show similar spectroscopic properties with an absorption maximum around 647 to 648 nm and an emission maximum between 660 and 670 nm. Curves are annotated with the corresponding particle diameter. UV–vis spectra were recorded using undiluted suspensions, whereas diluted suspensions were used to measure the fluorescence intensity.

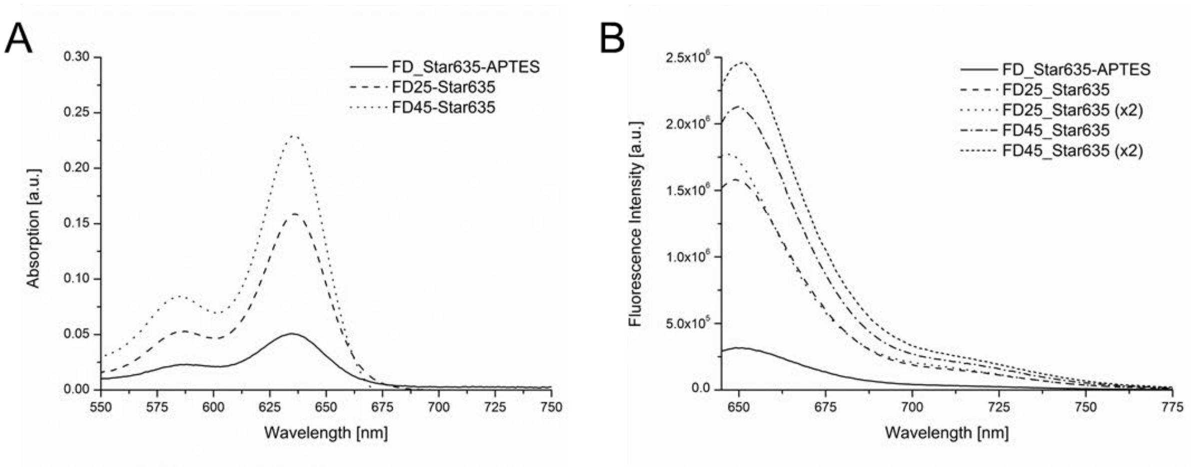

Figure S18: UV–vis (left) and fluorescence (right) spectra of silica nanoparticles obtained after *in situ* incorporation of modified Star635-APTES conjugate (FD_Star635-APTES) and Star635-CS-APTES conjugate (FD25_Star635 and FD45_Star635) into the matrix material. Neither the cysteic acid linker nor the concentration of the dye has a significant influence to the position of the absorption and emission maxima.

Table S19: Results of the leaching experiments of the Atto647N dyed nanoparticles. The dye leaching was determined by comparing the relative fluorescence intensity before and after centrifugation of the particle suspension through two different membranes with molecular weight cut off (MWCO) of 30 kDa and 100 kDa. The measurements revealed that there was nearly no dye leaching in the particle suspensions.

**SiO_2-NP**	**Membrane (MWCO: 100 kDa)** [%]	**Membrane (MWCO: 30 kDa)** [%]
FD15	1.8	0.8
FD25	6.5	2.7
FD45	6.6	2.7
FD60	4.8	2.3
FD80	4.5	4.5
Stoe15	13.8	6.7
Stoe60	33.7	24.4
CD	1.9	1.0

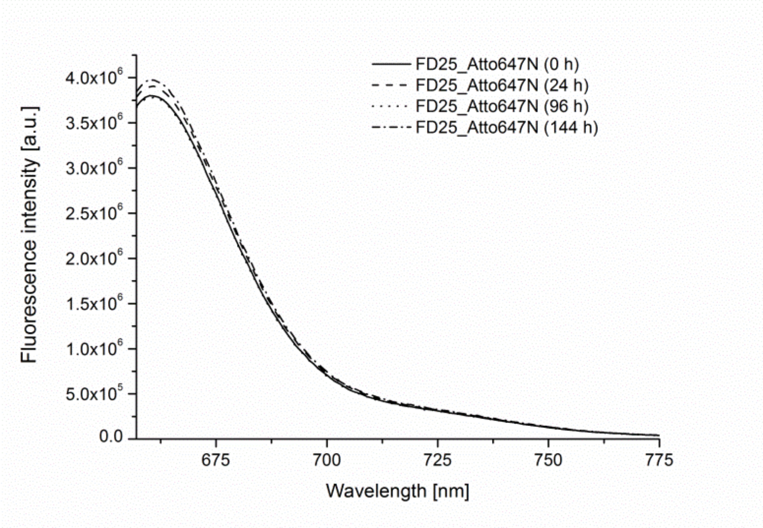

Figure S20: Fluorescence stability measurements of FD25_Atto647N over a total time period of 144 h. No change in the fluorescence intensity could be observed.

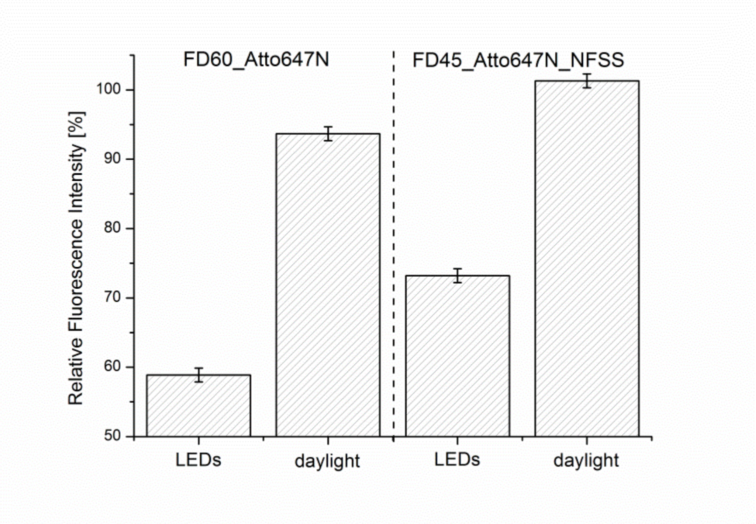

Figure S21: Results of the photobleaching experiments of FD45_Atto647N_NFSS and FD60_Atto647N, which were illuminated over a period of 20 min by the LEDs and daylight respectively.

7. Cell tests

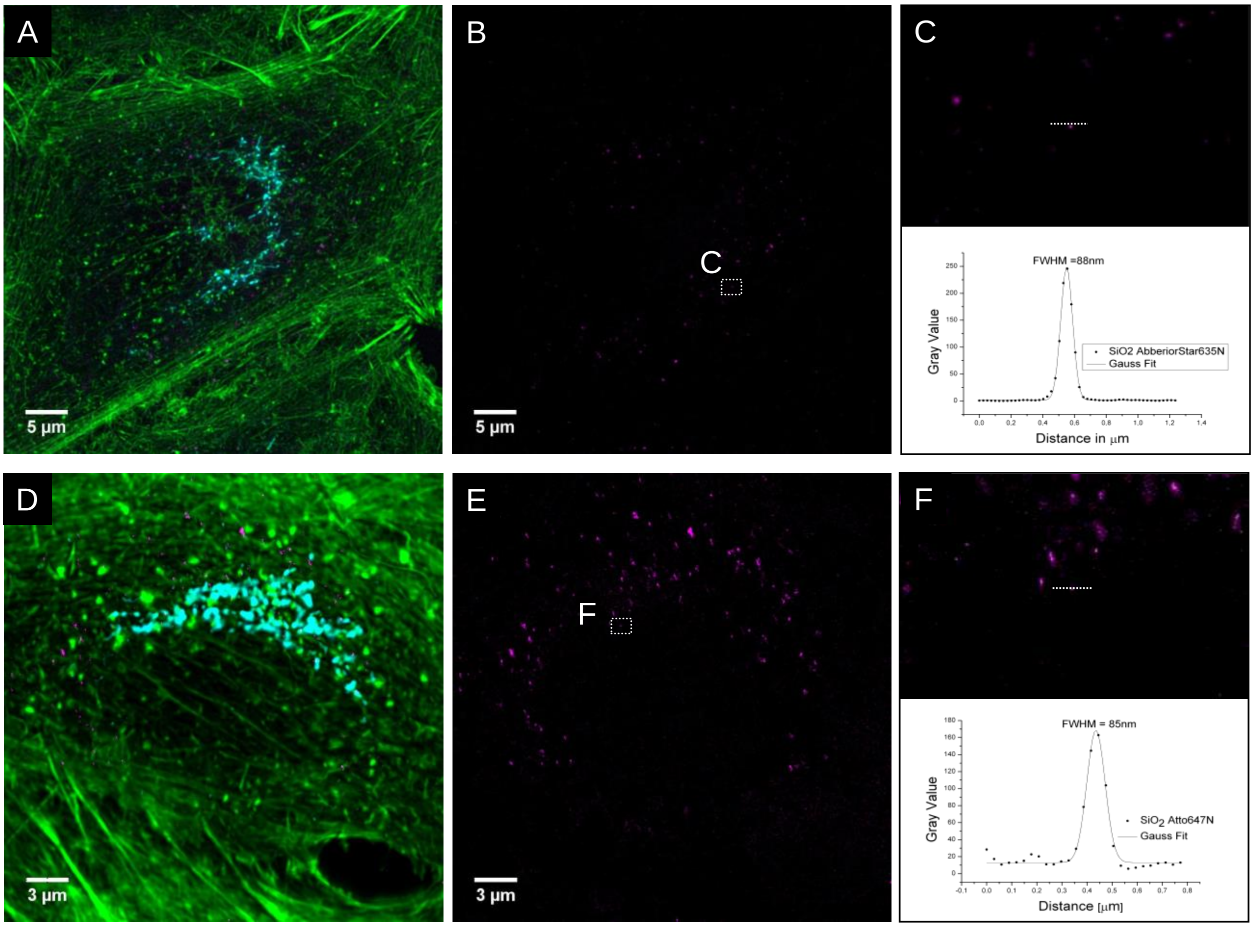

Figure S22: STED imaging of A549 cells incubated with 10 µg/mL FD25_Star635 (A) or FD25_Atto647N (D) for 24 h. The actin cytoskeleton is depicted in green, the cis-Golgi network in cyan and silica nanoparticles in magenta. Particles were imaged in STED mode (B and E), cellular structures in confocal mode and recorded z-stacks were deconvolved. C and F show an intensity plot through a sample nanoparticle (dotted line) within the cell. The full width of half maximum (FWHM) was 88 or 85 nm, which is below the classical optical resolution limit. Brightness and contrast of images was adjusted for better visualization using ImageJ.

References

[1] Hartlen, D.; Athanasopoulos, A. P. T.; Kitaev, V. *Langmuir* **2008**, *24*, 1714–1720. doi:10.1021/la7025285

[2] Stöber, W.; Fink, A.; Bohn, E. *J. Colloid Interface Sci.* **1968**, *26*, 62–69. doi:10.1016/0021-9797(68)90272-5

[3] Herz, E.; Ow, H.; Bonner, D.; Burns, A.; Wiesner, U. *J. Mater. Chem.* **2009**, *19*, 6341–634. doi:10.1039/b902286d
